# Browning affects pelagic productivity in northern lakes by surface water warming and carbon fertilization

**DOI:** 10.1111/gcb.16469

**Published:** 2022-10-19

**Authors:** Isolde C. Puts, Jenny Ask, Anne Deininger, Anders Jonsson, Jan Karlsson, Ann‐Kristin Bergström

**Affiliations:** ^1^ Climate Impacts Research Centre, Department of Ecology and Environmental Science Umeå University Umeå Sweden; ^2^ Umeå Marine Sciences Centre Umeå University Umeå Sweden; ^3^ Norwegian Institute for Water Research (NIVA) Oslo Norway

**Keywords:** acidification, bicarbonate system, DOC, inorganic carbon, primary production, stoichiometry, supersaturation, temperature

## Abstract

Global change impacts important environmental drivers for pelagic gross primary production (GPP) in northern lakes, such as temperature, light, nutrient, and inorganic carbon availability. Separate and/or synergistic impacts of these environmental drivers on pelagic GPP remain largely unresolved. Here, we assess key drivers of pelagic GPP by combining detailed depth profiles of summer pelagic GPP with environmental and climatic data across 45 small and shallow lakes across northern Sweden (20 boreal, 6 subarctic, and 19 arctic lakes). We found that across lakes summer pelagic GPP was strongest associated with lake water temperatures, lake carbon dioxide (CO_2_) concentrations impacted by lake water pH, and further moderated by dissolved organic carbon (DOC) concentrations influencing light and nutrient conditions. We further used this dataset to assess the extent of additional DOC‐induced warming of epilimnia (here named internal warming), which was especially pronounced in shallow lakes (decreasing 0.96°C for every decreasing m in average lake depth) and increased with higher concentrations of DOC. Additionally, the total pools and relative proportion of dissolved inorganic carbon and DOC, further influenced pelagic GPP with drivers differing slightly among the boreal, subarctic and Arctic biomes. Our study provides novel insights in that global change affects pelagic GPP in northern lakes not only by modifying the organic carbon cycle and light and nutrient conditions, but also through modifications of inorganic carbon supply and temperature. Considering the large‐scale impacts and similarities of global warming, browning and recovery from acidification of lakes at higher latitudes throughout the northern hemisphere, these changes are likely to operate on a global scale.

## Graphical abstract

Conceptual overview of what our results (text and arrows in red) add to the current consensus (text and arrows in black/gray) regarding drivers of summer pelagic GPP (green) across a gradient of increasing DOC concentrations. Our results point out that CO_2_ concentrations increasing along the DOC gradient are additional major drivers of summer pelagic GPP, that together with DOC:DIC stoichiometry further enhance GPP. Increased water temperatures with increasing DOC can promote GPP rates even more and thus partly counteract the negative impact of reduced light conditions caused by DOC on pelagic GPP.

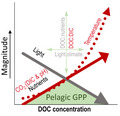

## 1 INTRODUCTION

Global environmental changes such as warming, recovery from acidification and forestry have altered the biogeochemistry in northern lakes (Creed et al., 2018; Skjelkvåle et al., 2001) and have thus impacted important environmental drivers for pelagic gross primary production (GPP). As phytoplankton are crucial providers of energy, minerals and biochemical compounds for higher consumers, understanding global change impacts on pelagic GPP is highly relevant for aquatic food webs in general (Müller‐Navarra, 2008; Peltomaa et al., 2017; Sterner & Hessen, 1994). At higher latitudes, lakes are particularly common (Lehner & Döll, 2004; Verpoorter et al., 2014) and surface air temperature anomalies related to climate change are the greatest (the arctic amplification) (Cohen et al., 2014; Hansen et al., 2010; Serreze et al., 2009), emphasizing the need of understanding global change impacts on northern lakes in particular. Increased air temperatures induce a direct warming of surface waters (O'Reilly et al., 2015; Schneider et al., 2009), but also indirectly increase precipitation (De Wit et al., 2016; Hudson et al., 2003; Lind & Kjellström, 2008). Warming and increased precipitation further promote enhanced catchment vegetation cover (i.e., greening), which together with forestry and recovery from acidification induce enhanced loadings of terrestrial dissolved organic material (DOM) to northern lakes (Creed et al., 2018; Finstad et al., 2016; Kritzberg, 2017). Important components of DOM related to lake biogeochemistry are nutrients and colored dissolved organic carbon (DOC). However, neither the separate nor the synergistic impacts of large‐scale changes of warming and altered lake biogeochemistry on pelagic GPP at higher latitudes are resolved.

**FIGURE 1:**
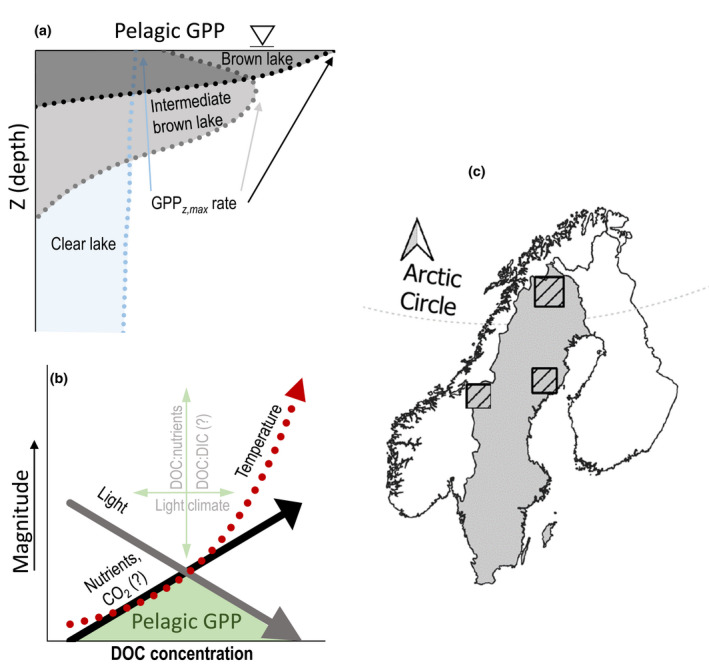
(a) Conceptual figure showing depth profiles of GPP_z_ rates in a stratified brown lake high in DOC (black), in a lake intermediate in DOC (gray) and in a clear lake low in DOC (blue). In brown lakes GPP is confined to a shallower epilimnion, but rates are high, and in clear lakes GPP is spread deeper in the water column, but with consistently lower GPP_*z*_ rates. (b) Conceptual figure of pelagic GPP_lake‐average_ distribution (green) with increasing DOC concentrations, initially limited by nutrients, and at higher DOC concentrations by light inhibition. At higher DOC concentrations increased temperatures (red arrow) can increase maximum GPP_z_ rates and could thus to some extent counteract the negative impact of reduced light on pelagic GPP. The height of the peak in GPP is defined by DOC:nutrient stoichiometry, and the location of the GPP peak by the coloring of DOC. (c) Sampling locations, with the lakes from the Arctic biome in the northernmost outlined area, subarctic in the western outlined area, and boreal in the southernmost outlined area. Map lines delineate study areas and do not necessarily depict accepted national boundaries.

Pelagic GPP is commonly measured at discrete depths (here GPP_*z*_ rates) resulting in differently shaped depth profiles depending on light availability and the maximum GPP rate (here: GPP_z,max_, per m^3^) occurs where optimal growth conditions are present (Wetzel & Likens, 1991; Figure 1a). The GPP_*z*_ rates can be upscaled to a lake‐average (GPP_lake‐average_, per m^2^) by integrating rates over the water column and dividing them by the lake surface area. Key environmental drivers therefore likely differ between GPP_z,max_ rates that depend on local conditions and GPP_lake‐average_, which reflects the response to integrated environmental conditions.

The pelagic GPP_z_ rates can be constrained by dissolved inorganic carbon (DIC), nutrients, light (energy), and temperature (Graham et al., n.d.; Wetzel & Likens, 1991). Carbon dioxide (CO_2_) is the most bioavailable DIC source used in photosynthesis by phytoplankton, and pH regulates the amount of CO_2_ relative to (bi)carbonates in lake water DIC (Huisman et al., 2018; Wetzel & Likens, 1991). Hence, lake pH is important for GPP via its effect on the relative amount of CO_2_. The degree of CO_2_ supersaturation of surface waters often increases with DOM concentrations in lakes (Del Giorgio et al., 1999; Larsen et al., 2011; Sobek et al., 2003). Although several mechanisms can cause CO_2_ supersaturation in lakes, terrestrial DOM is important as it is correlated with lower pH in lakes and its mineralization generates CO_2_ (Lazzarino et al., 2009; Nydahl et al., 2020; Stets et al., 2017). DIC and CO_2_ can have a positive effect on pelagic GPP, so called “carbon fertilization” (Hammer, 2019; Jansson et al., 2012; Kragh & Sand‐Jensen, 2018). However, nutrient (Jones, 1992; Klug, 2002; Roulet & Moore, 2006) and light availability (Carpenter et al., 1998; Jones, 1992) have been focus points when assessing the role of DOM for pelagic GPP (rates and lake‐averages) in northern lakes.

Several empirical and modeling studies show that the tradeoff between light and nutrient availability promotes a unimodal distribution of pelagic GPP_lake‐average_ with increased lake DOC (Rivera Vasconcelos et al., 2018; Solomon et al., 2015), where the peak in GPP_lake‐average_ is determined by the DOC:nutrient stoichiometry, whereas the location of the GPP peak along the DOC axis is determined by the light climate (Bergström & Karlsson, 2019; Kelly et al., 2018). Nonetheless, the unimodal relationships vary and are not always observed, suggesting that other factors influence this relationship and regulate pelagic GPP_lake‐average_ (Kelly et al., 2018). For example, co‐limitation by CO_2_ and nutrient on pelagic GPP has been demonstrated in experimental studies (Low‐Décarie et al., 2011, 2014), and field studies in northern oligotrophic lakes (Brown et al., 2019; Hamdan et al., 2018; Jansson et al., 2012). In addition, results from controlled experimental pond ecosystems further suggest that warming alone may additionally amplify pelagic GPP_lake‐average_ at all levels of lake water DOC concentrations (Hamdan et al., 2018; Figure 1b). Increased colored DOC also results in epilimnion warming, especially in small and shallow lakes, likely through intensified water column stratification (Bartosiewicz et al., 2016, 2019; Houser, 2006; Pilla et al., 2018, 2020). Since a warmer climate promotes higher lake DOC concentrations via indirect effects occurring in the lakes catchment (Laudon et al., 2012; Tetzlaff et al., 2013), increasing air temperatures can amplify the epilimnion warming via increasing DOC, that is, additional DOC‐induced warming of the lake epilimnia (here named: internal warming). However, intrinsic effects of temperature on pelagic GPP_lake‐average_ may be hard to disentangle from colored DOC and nutrients (Bergström et al., 2013; Faithfull et al., 2011; Klug, 2005), and CO_2_ (Jansson et al., 2012; Jonsson et al., 2001) as they are tightly correlated, making temperature redundant in models that include DOC, nutrients, and CO_2_.

Moreover, these recognized patterns and relationships between environmental drivers of pelagic GPP (rates and lake‐average) may not be consistent across biomes and over seasons due to variable DOC:nutrient stoichiometry, coloring of the DOC, differences in climate, catchment properties, air temperatures, and light conditions (Bergström & Karlsson, 2019; Isles et al., 2021; Kelly et al., 2018; Seekell et al., 2015). Overall, several studies have assessed how DOC impacts light and nutrient availability and quality, lake water DIC concentrations, and temperature (summarized in Figure 1b), but the relative contribution of DIC, CO_2_, temperature, and DOC:DIC stoichiometry as additional drivers for pelagic GPP_lake‐average_ has been far less assessed in empirical studies.

Here, we investigate how global change by its impact on key environmental drivers affects summer pelagic GPP in northern lakes over a large spatial scale. For this reason, we collected data on pelagic GPP depth profiles and GPP_lake‐averages_, together with physico‐chemical lake parameters in summer (June–August) from 45 lakes in northern Sweden. The lakes were spread over three different biomes (20 boreal, 6 subarctic, and 19 Arctic lakes; Figure 1c), covering a colored DOC gradient from 1.0 to 19.5 mg L^−1^. Besides abiotic in situ conditions and lake bathymetry, we also investigate the additional effects of air temperature on the DOC‐induced warming of the lake epilimnia (i.e., internal warming), and how the inorganic carbon sources (CO_2_ vs. DIC) are a function of lake water pH. We assess how key environmental drivers related to global change affect summer GPP (rates and lake‐averages) both across northern Sweden and per biome, and how DOC:nutrient (DOC:TN; DOC:TP) and DOC:DIC stoichiometry, and lake water temperature influence GPP_lake‐average_. We hypothesize that: (1) Internal warming of lakes increases along the DOC gradient, (2) GPP_z,rates_ relates to temperature, DOC, nutrients DOC:nutrient stoichiometry and CO_2_, and GPP_lake‐average_ relates to similar drivers but mostly to lake bathymetry and (3) drivers are biome specific.

## 2 METHODS

### 2.1 Study area, sampling and data compilation

We compiled data of pelagic GPP, together with water chemistry and bathymetry, from 45 small and shallow lakes (lake surface area between 0.6 and 9.4 ha, and depth between 3.7 and 15.8 m) in northern Sweden spread over Swedish boreal (20 lakes), subarctic (6 lakes), and Arctic (19 lakes) biomes (Figure 1c). These biomes include boreal forests, subarctic and Arctic (alpine) tundra biomes and are classified according to AMAP (1998). Lakes in the boreal were smaller than lakes in the other biomes (mean lake surface area: boreal 3.4 ± 2.3, subarctic 7.0 ± 3.7, Arctic 8.3 ± 5.2 ha), and boreal lakes (233–366 m above sea level [m.a.s.l.]) were located at lower altitudes than the mountainous subarctic (578–655 m.a.s.l.) and Arctic lakes (270–1115 m.a.s.l.). Lakes in the boreal biome were located in boreal coniferous forested areas, and lakes in the subarctic were surrounded by wetlands and open areas with relatively sparse (mainly deciduous/birch and willow) vegetation. Lakes in the Arctic covered a wide range of altitudes (altitude 270–1140 m.a.s.l.), both above and below the tree line, and were characterized by open areas above the tree line, or sparsely vegetated with deciduous shrubs below the tree line. Anthropogenic influences such as agriculture, urbanization, and forestry on the lakes are minor. All lakes were sampled between 21 June and 5 August, in variable years (years 1999–2019; see Figure S1). Data and sampling methods of pelagic GPP and water chemistry for 35 of the lakes are described in detail in earlier publications (Ask et al., 2009; Deininger et al., 2017, 2019; Karlsson et al., 2001), and the remaining 10 lakes (four in the boreal and six lakes in the subarctic biome, original to this study) are sampled using similar techniques. The data can be found at https://doi.org/10.5061/dryad.t1g1jwt5k.

### 2.2 Pelagic GPP

Pelagic GPP was measured in situ at the surface and at subsequent 1 m depth intervals, with additional measurements at 0.25 and 0.5 m, where the deepest measurement depended on the lake depth and water turbidity (sensu Ask et al., 2009; Deininger et al., 2017, 2019; Karlsson et al., 2001). Measurements were done by incubating (single or replicates, see raw data) transparent glass bottles filled with water from the sampling depth, with additional incubations in dark bottles at the most shallow and deepest measurements, for about 4 h midday using ^14^C isotopic tracer as described by Schindler et al. (1972). We used raw isotopic activity values from all datasets, and recalculated GPP values similarly for consistency among the datasets. We further estimated the consumption of the DIC pool in the bottles to rule out that DIC became limiting during the 4 h incubation by calculating the DIC consumption from the GPP_z max_ in the lake over the incubation time of 4 h in relation to the DIC pool in the incubation bottle. This showed that in all cases the consumption of DIC in the bottle was less than 5% of the DIC pool (median value 0.3%), except for Struptjärn (which we excluded from the analyses) where the consumption was clearly higher and ca 62% of the pool in the bottle was consumed. Calculated GPP values were extrapolated to daily GPP using the ratio of incident photosynthetically active radiation (PAR) during incubation in relation to daily PAR. GPP_z,max_ rates represent the maximum rate measured over the water column occurring at one specific depth in each lake (i.e., the peak in the vertical distribution; Figure 1a). GPP rates measured at discrete depths (GPP_*z*_ rates) were upscaled to a single average GPP estimate per lake (GPP_lake‐average_) by integrating the rates over the water column and dividing them by the lake surface area (Table S1). We used volume‐weighted pelagic GPP_*z*_ rates when upscaling to GPP_lake‐average_.

### 2.3 Water chemistry

Since most of the pelagic GPP takes place in the epilimnion, and nutrients within the epilimnion are well mixed (all lakes stratified except for nine Arctic lakes: see raw data), we only used values from the epilimnion (i.e., 1 m) when relating water chemistry concentrations to pelagic GPP_z,max_ rates and GPP_lake‐average_ (see earlier publications for details on sampling procedures). Samples for pH, DOC, DIC, total nitrogen (TN), and phosphorous (TP) were thus taken at 1 m depth (epilimnion) or were taken from composite water samples (lakes from Ask et al. (2009)). In short, DOC was filtered through a 0.45 μm filter (Sarstedt Filtropur), acidified using HCl to an end concentration of 12 mM, and stored in a refrigerator before analyzed. TN and TP (unfiltered) samples were kept frozen until analysis. Dissolved inorganic carbon (DIC) samples were taken by injecting 4 ml (air free) lake water into a tightly sealed 18 ml glass vial (pre‐flushed with N_2_) containing 0.1 ml 1.2 M HCl and analyzed as soon as possible. Specific laboratory operating procedures afterwards can be found in Ask et al. (2009), Deininger et al. (2017, 2019) and Karlsson et al. (2001), or in Puts et al. (2022) for the previously unpublished lakes. We estimated DOC:nutrient and DOC:DIC ratios (by weight) and we log‐transformed DOC:TP, DOC:TN, and DOC:DIC ratios (Isles, 2020). pH was measured immediately after sampling in the laboratory, and CO_2_ concentrations in the lake water were calculated from DIC, pH, and temperature, following guidelines from the Water Quality Analysis Simulation Program (WASP) by the United States Environmental Protection Agency (EPA) (https://www.epa.gov/sites/default/files/2018‐05/documents/wasp‐ph‐release‐notes.pdf).

### 2.4 Light, temperature, and bathymetry

PAR and temperature in all lakes were measured using a handheld probe at every meter throughout the water column at the deepest part of the lake, with additional measurements at 0.25 and 0.5 m. Light attenuation coefficients (Kd) were calculated as the absolute slope of natural logarithmically transformed PAR against depth. From the Kd we calculated the percent of incoming light at the depth where the pelagic GPP_z,max_ was located (%light) relative to the surface (100%), and for each lake we calculated the euphotic depth (the depth where 1% of surface light remains; z_euph_). Together with incoming daily PAR (defined as PAR) we also calculated the daily PAR at depth for the GPP_z,max_ rates (PAR_depth_). Daily PAR was collected from stations located next to the lake, or acquired from open databases (Laudon et al., 2021; SMHI, website). Pelagic GPP_z,max_ rates are related to their depth specific temperature (*T*_depth_), whereas pelagic GPP_lake‐average_ estimates are related to temperature at a fixed epilimnion depth of 0.2 m (*T*_water_). Average air temperatures (*T*_air_) are obtained using monthly air temperature averages 1 month before sampling from weather stations located within a maximum range of 60 km from the sampling sites (data extracted from the Swedish Meteorological and Hydrological Institute [https://www.smhi.se/klimat/klimatet‐da‐och‐nu]), including a temperature decrease of 0.57°C per 100 m elevation difference between station and sampling site (sensu Karlsson et al., 2005 and references therein). Internal warming of the epilimnion is here defined as *T*_water_–*T*_air_. We calculated the lake‐average depth (z_avg_) and volumes (as a whole, or in different sections) and lake surface areas (Area) from detailed bathymetry (Table S1).

### 2.5 Statistical analyses

Differences in water chemistry variables among the biomes are tested in ANOVAs with estimated marginal means compared per biome (Searle et al., 1980). To relate internal warming to DOC concentrations, we first selected which curve type (linear, logarithmic, or exponential) best explained (highest *R* ^2^) *T*_air_ and *T*_water_ versus DOC, and then identified the main drivers of internal warming using a multiple linear regression (MLR) with forward selection that included the variables DOC, Kd, Area, z_max_, z_avg_, z_euph_, and lake altitude. The significance of internal warming against DOC was tested with regression analyses.

We investigated which drivers explain GPP (GPP_z,max_ rates and GPP_lake‐average_) best throughout northern Sweden (i.e., including all lakes) in an MLR with forward selection. We tested data for underlying assumptions of parametric tests, but data were not corrected for collinearity. To overcome collinearity issues, unequal sample sizes per biome, we further investigated the spread of explanatory variables and GPP in a PLS using the plsr package (Mevik & Wehrens, 2007) in R, again both including data from all lakes and in addition per biome. A PLS is a comparable method to the more well‐known principal component analysis (PCA), but specifically suitable for datasets with high predictor values compared to observations, like our dataset. VIP scores are a measure of how substantially the variable adds to the model, and the loading describes the correlation intensity between the variable and the predictor. We considered a VIP <0.9 as minimum value for a variable to substantially add to the model (Mehmood et al., 2012). Included variables for both GPP_z,max_ rates and GPP_lake‐average_ are presented in Table 1, and we included similar variables in the MLR and the PLS.

**TABLE 1:** Multiple linear regressions with forward selection of (I) Internal warming, (II) Pelagic GPP_z,max_ rates and (III) Pelagic GPP_lake‐average_ with different included variables

Coefficients of explanatory variables	Intercept	*R* ^2^	df2	*F*	AIC	Deselected variables
(I) Internal warming (*T*_water_–*T*_air_) (*n* = 42)						
−0.692·z_avg_ (*)	7.498	0.193	41	9.8	68.1	DOC, Kd, Area, z_max_, z_euph_, Alt.
−0.708·z_avg_ − 0.003·Alt.(*)	8.909	0.273	40	7.7	65.5	DOC, Kd, Area, z_max_, z_euph_
(II) Pelagic GPP_z,max_ rates ^a^ (*n* = 42)						
1.023·CO_2_ (**)	0.663	0.562	40	51.3	−45.4	DOC, DIC, TN, TP, T_depth_, PAR_depth_
0.849·CO_2_ + 0.102·T_depth_ (**)	−0.995	0.758	39	60.9	−68.3	DOC, DIC, TN, TP, PAR_depth_
(III) Pelagic GPP_lake‐average_^a^ (*n* = 42)						
0.921·CO_2_ (**)	0.766	0.527	40	45.6	−46.3	DIC, TN, TP, PAR, Area, z_max_, z_avg_, z_euph_, *T*_water_, *T*_air_, DOC:TN^a^, DOC:TP^a^, DOC:DIC^a^ DOC:CO_2_^a^
0.769·CO_2_ + 0.120· T_air_ (**)	−0.602	0.667	39	40.0	−59.3	DIC, TN, TP, PAR, Area, z_max_, z_avg_, z_euph_, DOC:TN^a^, DOC:TP^a^, DOC:DIC^a^ DOC:CO_2_^a^
0.738·CO_2_ + 0.139·T_air_ − 0.630·DOC:DIC^a^ (**)	−0.368	0.719	38	33.3	−64.7	DIC, TN, TP, PAR, Area, z_max_, z_avg_, z_euph_, DOC:TN^a^, DOC:TP^a^, DOC:CO_2_^a^

*Note*: Variables marked with ^a^ are log‐transformed and abbreviations are as follows: z_avg_ = average lake depth (m), Area = lake surface area (hectare), z_max_ = maximum lake depth (m), z_euph_ = euphotic depth (m), Alt. = lake altitude (m), CO_2_ = carbon dioxide in lake water (mg·L^−1^), DOC = dissolved organic carbon (mg·L^−1^), DIC = dissolved inorganic carbon (mg·L^−1^), TN = total nitrogen (mg·L^−1^), TP = total phosphorus (μg·L^−1^), T_depth_ = temperature at depth (°C), *T*_water_ = temperature at 0.2 m (°C), *T*_air_ = average air temperature of the previous month (°C), PAR_depth_ = daily incoming PAR at depth (W·m^−2^), PAR = daily incoming PAR at surface (W·m^−2^), df2 = degrees of freedom (denominator), AIC = Akaike's information criterion. Levels of significance of the models are indicated as follows: *p < .05, **p < .001.

Lake 13 (no *T*_water_ data) and lake 15 (ice on lake) were removed from the dataset assessing internal warming. We removed these two lakes, together with lake 4 (DIC values two standard deviations above averages), and Struptjärn (GPP_z,max_ and GPP_lake‐average_ two standard deviations above averages because of an invasive G. *semen* bloom: see Deininger et al., 2019) from the dataset including GPP. GPP_z,max_ rates and GPP_lake‐averages_ were log‐transformed to meet conditions for performing parametric tests. The quality of the multiple linear regressions models was tested using Akaike's information criterion (AIC). We verified that the warming pattern is statistically independent of the sampling year and timeframe (Figure S1). Statistical analyses were conducted in IBM spss statistics v. 26, and in R. We considered an effect statistically significant at *p* < .05 (two‐tailed for the ANOVAs and MLR).

## 3 RESULTS

### 3.1 Water chemistry and internal warming

Our study lakes cover a wide range of DOC, DIC, and nutrient concentrations (Table S2). DOC (boreal: 3.8–19.5, subarctic: 4.4–7.2, Arctic: 1.0–13.1 mg·L^−1^) and TN (boreal: 251–532, subarctic: 143–199, Arctic: 80‐551 μg·L^−1^) concentrations were higher in the boreal lakes than in the other lake regions (*p* < .001 and *p* < .01, respectively; Table S3 for statistics), whereas TP concentrations (boreal: 4.0–33.9, subarctic: 3.7–5.2, Arctic: 2.6–17.1 μg·L^−1^) were lower in subarctic lakes compared to lakes in the other regions (*p* < .05). Lake DIC concentrations (boreal: 0.7–4.2, subarctic: 1.0–2.6, Arctic: 0.1–3.4 mg·L^−1^) did not differ among regions, whereas pH values in the Arctic lakes (6.1–7.8) were higher than in the boreal (4.8–7.0) and subarctic (5.7–6.0) lakes (*p* < .001 performed on equivalence values). CO_2_ values (boreal 0.1–2.2, subarctic: 0.7–1.9, Arctic 0.0–0.62 mg·L^−1^) were lower in the Arctic compared to the other biomes (*p* < .01). Kd values (boreal 0.4–4.2, subarctic: 0.9–1.4, Arctic 0.2–1.4 m^−1^) were higher in boreal lakes compared to lakes in the other biomes (*p* < .001). Air temperatures (T_air_; boreal: 12.3–16.6, subarctic 10.2–10.6, Arctic 6.4–12.7°C) were higher in the boreal than in the subarctic and Arctic biomes (p < 0.001), and lake water temperatures at 0.2 m (*T*_water_; boreal: 15.8–23.0, subarctic 16.5–18.7, Arctic 0.3–23.0°C) were lower in the Arctic lakes compared to in lakes in the other regions (*p* < .001).

**FIGURE 2:**
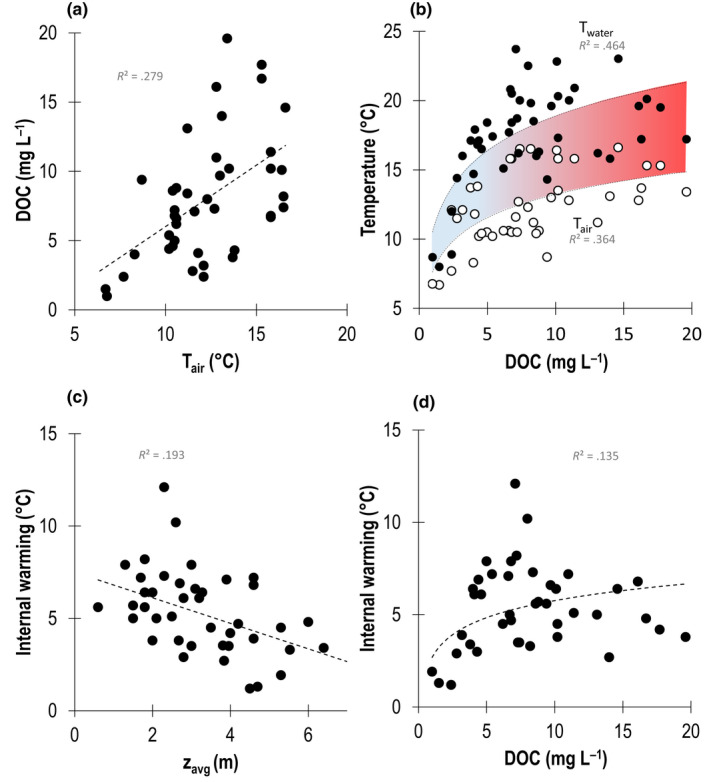
(a) Climate gradients visualized by plotting dissolved organic carbon (DOC, mg·L^−1^) against monthly average air temperature (T_air_ in °C). (b) Internal warming (blue to red colored), defined as the difference between the logarithmic functions of T_air_ (white dots) and water temperature at 0.2 m depth (T_water_ in °C, black dots), plotted against DOC. (c) Internal warming (°C) of the lakes as a function of average lake depth (m). (d) Internal warming (°C) of the lakes as a function of DOC. All correlations displayed have a *p* < .05 (two‐tailed).

DOC concentrations increased with T_air_ (Figure 2a; *p* < .000). The relationships between temperature (in water and air) and DOC were best explained by a log‐linear regression (*R* ^2^ = .46 and *R* ^2^ = .36, respectively), resulting in an initially increasing but eventually dampened internal warming (*T*_water_–*T*_air_) with increasing DOC concentrations (Figure 2b; *p* < .05). Internal warming of the epilimnion was best explained (*R* ^2^ = .19) by average lake depth (z_avg_), and, thus, showed a declining trend with increased average lake depth (Figure 2c; Table 1: regression I *p* < .01). The internal warming also increased with increasing DOC concentrations (Figure 2d: *p* < .05).

### 3.2 Summer pelagic GPP

**FIGURE 3:**
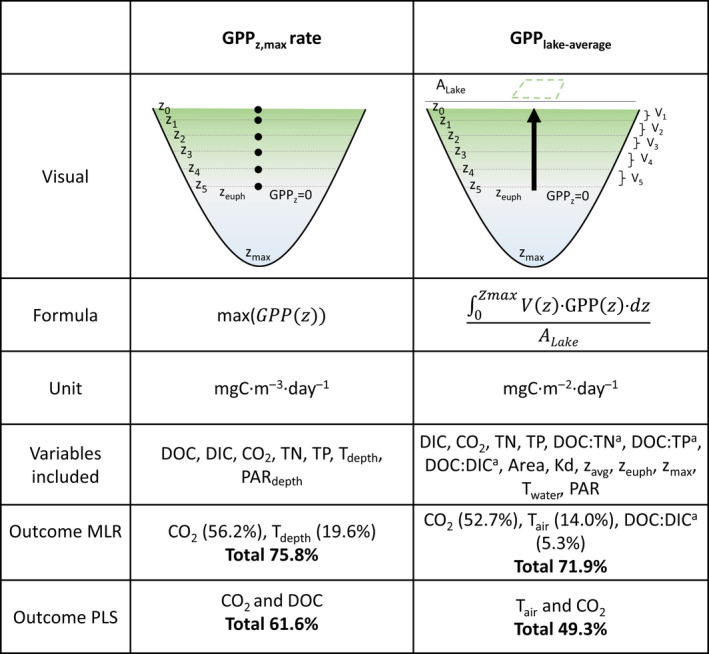
Graphical and mathematical visualization of GPP_z,max_ rates and GPP_lake‐average_ with their units, and included variables and outcomes of both the multiple linear regression (MLR) and partial least squares regression (PLS). Variables marked with ^a^ are log‐transformed and abbreviations are as follows: Area, lake surface area (hectare); DOC, dissolved organic carbon (mg·L^−1^); DIC, dissolved inorganic carbon (mg·L^−1^); PAR, daily incoming PAR at surface (W·m^−2^); PAR_depth_, daily incoming PAR at depth (W·m^−2^); T_air_, average air temperature of the previous month (°C); T_depth_, temperature at depth (°C); T_water_, temperature at 0.2 m depth (°C); TN, total nitrogen (μg·L^−1^); TP, total phosphorus (μg·L^−1^); z_avg_, average lake depth (m); z_euph_, euphotic depth (m); z_max_, maximum lake depth (m).

**FIGURE 4:**
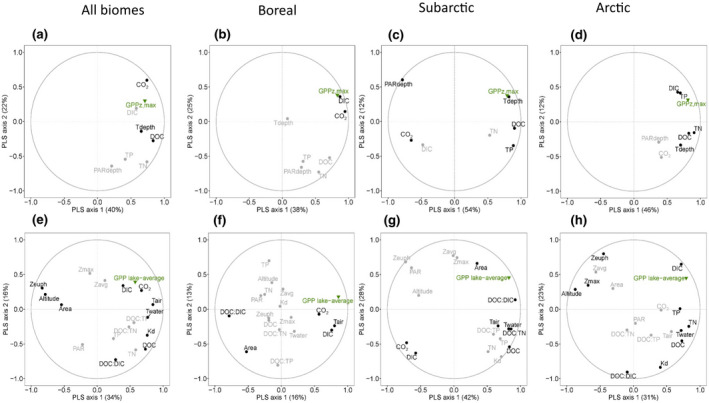
Partial least squares (PLS) regression biplots of environmental variables and (a–d) pelagic GPP_z,max_ rates and (e–h) GPP_lake‐average_. The biplots include (a, e) all lakes, and lakes from the (b, f) boreal, (c, g) subarctic, and (d, h) Arctic biome. Variables adding substantially to the PLS model with a VIP >0.9 are plotted in black, and variables that do not substantially add to the model with a VIP <0.9 are plotted in gray.

Pelagic GPP_z,max_ rates including lakes from all biomes increased with and were best explained by CO_2_ (56.2%) and water temperature at depth (additional 19.6%) in the MLR (Figure 3; Table 1: Regression II). These results were confirmed in the PLS regression including lakes from all biomes, as CO_2_ and DOC explained the variance in pelagic GPP_z,max_ rates best in both the first component (39.6%) and the second component (22.0%) (Figure 4a; Table S4). Variables that were included in the model that did not substantially contribute to any of the two components (VIP <0.9; Table S4) are shown in gray in the PLS figures (Figure 4). However, different drivers of pelagic GPP_z,max_ rates were selected per biome (Figure 4b–d). In the boreal lakes, CO_2_ and DIC were selected as best drivers explaining the variance in pelagic GPP_z,max_ rates for both the first component (38.0%), and the second component (24.6%) (Figure 4b). In the subarctic lakes, T_depth_ together with DOC explained both the first component (54.3%), and the second component (12.1%) best (Figure 4c). In the Arctic lakes, DOC and DIC were identified as drivers for the first component (46.0%), and also explained the second component (12.4%) best (Figure 4d).

Summer pelagic GPP_lake‐average_ including data from all biomes was best explained by CO_2_ (52.7%), T_air_ (additional 14.0%), and DOC:DIC (additional 5.3%) (Figure 3; Table 1: Regression III). Results from the PLS regression including all lakes were slightly different, with T_air_ and CO_2_ best explaining the variance in pelagic GPP_lake‐average_ for both the first component (33.6%) and the second component (15.7%) (Figure 4e; Table S4b). Although variables were quite spread out in the PLS plot, indicating slight correlation among variables, *T*_air_ was tightly coupled to *T*_water_ in the first component, as were lake altitude and lake surface area but to a lesser extent. The PLS plot revealed that within the first component, Kd was clustered with the drivers *T*_air_, *T*_water_, and nutrients (DOC, TN, TP, and DOC:nutrient stoichiometry to a lesser extent), whereas lake area and altitude had a negative impact and thus were negatively correlated with GPP_lake‐average_ (Figure 4e; Table 2). Moreover, both euphotic depth, lake surface area, and lake altitude had a high VIP score (1.3, 1.3, and 1.1, respectively), indicating that these variables contributed substantially to the model, and thus explained part of the variance in pelagic GPP_lake‐average_. The PLS plots also visualize that nutrients and DOC:nutrient stoichiometry did not substantially add to the model best explaining GPP_lake‐average_, and that they were clustering together against the other variables.

**TABLE 2:** Overview of the selected variables explaining the first and second component in the PLS, including all lakes and per biome

Biome/GPP	All lakes	Boreal	Subarctic	Arctic
GPP_z,max_	CO_2_, **DOC**	CO_2_, DIC	T_depth_, **DOC**	DIC, **DOC**
GPP_lake‐average_	T_air_, CO_2_	DOC:DIC, **T**_**air**_	DOC:DIC, *CO*_*2*_	DIC, TP

*Note*: Selected variables with negative loadings are in italic font, variables in bold are alternating positive or negative in their loadings within the first and second component.

**FIGURE 5:**
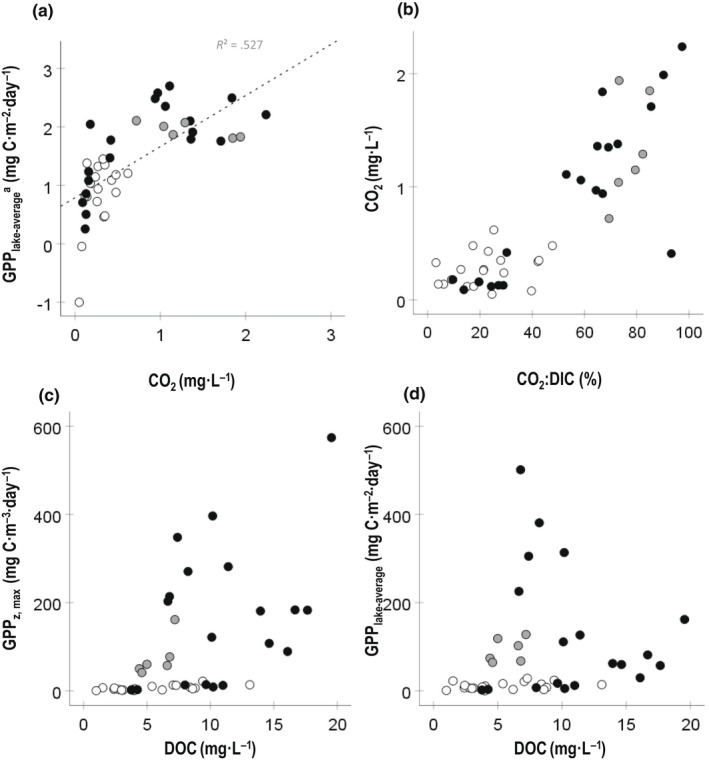
(a) GPP_lake‐average_ (log‐transformed) against water CO_2_ concentrations (mg·L^−1^) per biome, (b) water CO_2_ concentrations as a function of the molar ratio in percent of total pool of dissolved organic carbon (CO_2_:DIC [%]), and (c) GPP_z,max_ rates and (d) GPP_lake‐average_ against DOC concentrations (mg·L^−1^). Data include lakes from the boreal (black), subarctic (gray) and Arctic biome (white). GPP_lake‐average_ values are expressed in mgC·m^−2^·day^−1^, and GPP_z,max_ rates in mgC·m^−3^·day^−1^.

Also, for pelagic GPP_lake‐average_, different drivers were selected per biome (Figure 4f–h). For the boreal lakes, DOC:DIC and T_air_ were selected as variables best explaining the variance in pelagic GPP_lake‐average_ in both the first component (16.4%), and the second component (13.1%) (Figure 4f). For the subarctic lakes, DOC:DIC and CO_2_ explained the first component (42.1%), and DIC and CO_2_ explained the second component (28.0%) best (Figure 4g). For the Arctic lakes, DIC and TP were identified for both the first component (31.3%) and the second component (22.8%) (Figure 4h). GPP_lake‐average_ increased with CO_2_ in the boreal and Arctic biome but had a decreasing trend in the subarctic (Figure 5a; negative loadings in PLS: Table S4b). Moreover, the fraction of DIC that is CO_2_ was very variable among our lakes and differed significantly (*p* < .05) between the Arctic and boreal and was on average lowest in the Arctic (22.7%), followed by the boreal (52.8%) and then by the subarctic biome (77.1%; Figure 5b).

### 3.3 Nutrient stoichiometry and pelagic GPP

**FIGURE 6:**
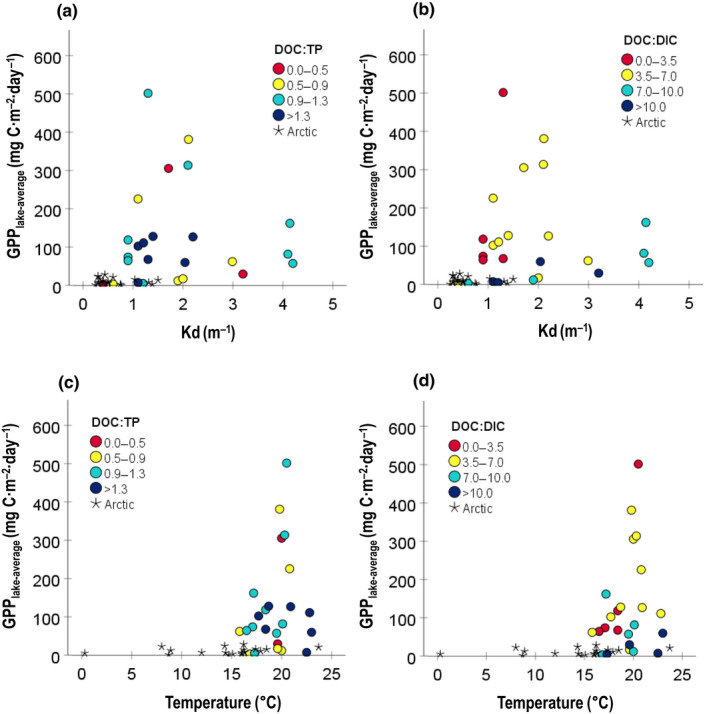
GPP_lake_ _‐average_ plotted in different categories of (a, c) DOC:TP ratios and (b, d) DOC:DIC ratios, against (a, b) Kd (m^−1^), and (c, d) water temperature at 0.2 m depth (°C). Lakes from the Arctic biome are plotted as a separate category (star shaped).

GPP_z,max_ rates increased with DOC (Figure 5c), whereas the pelagic GPP_lake‐average_ tended to be unimodally related with DOC, and most lakes had DOC concentrations below the GPP peak occurring at DOC concentrations around 9 mg·L^−1^ (Figure 5d). We also investigated if DOC:nutrient stoichiometry (here DOC:TN, DOC:TP) and DOC:DIC modified the GPP_lake‐average_ relationship with DOC and categorized these into different levels similar to the procedure in Kelly et al. (2018) for DOC:TP (Figure 6). As GPP in the Arctic was clearly lower compared to the other biomes, we marked GPP in the arctic biome as a category of its own. To eliminate the effect of variable coloring of DOC on a lake's light climate, we used Kd instead of DOC when graphically referring to the DOC gradient among lakes (see Figure S2 for relationship between DOC and Kd). For small and shallow lakes the light extinction coefficient (Kd) represents a good proxy for light availability in the mixed layer (Jones, 1998; Kelly et al., 2018). Generally, GPP_lake‐average_ followed a unimodal distribution with Kd, where higher DOC:TP and DOC:DIC ratios were related to lower GPP_lake‐average_ (Figure 6a,b; for the relationship with DOC:TN see Figure S3). Our dataset was, however, not large enough to perform statistical analyses on the different stoichiometry categories. Interestingly, the relationship between GPP_lake‐average_ and the different DOC:nutrient and DOC:DIC categories were affected by *T*_water_, and GPP_lake‐average_ showed a clear peak at *T*_water_ temperatures around 20°C (Figure 6c,d; Figure S3b). Again, our dataset was too small here to make statistical inferences.

## 4 DISCUSSION

In our study, we found empirical support for elevated warming of browner surface waters, especially in shallow lakes, and for that CO_2_ availability directed by a wide range of lake water pH is an important driver of pelagic summer GPP in northern lakes. Our results point out that global warming and increases in colored DOC may affect summer pelagic GPP, not only through changes in light and nutrient availability, but also via effects on water temperature and CO_2_ availability.

### 4.1 Internal warming of lake water

The internal warming (*T*_water_–*T*_air_) of surface waters was magnified at low to intermediate DOC concentrations, but was dampened at DOC concentrations >14.0 mg·L^−1^ (Figure 2) and decreased with average lake depth (z_avg_). More specifically, internal warming decreased with 0.96°C for every increasing m in average lake depth, with an internal warming being >5.0°C in lakes with z_avg_ <4 m (Table 1: Regression I; Figure 2c). This suggests that increasing air temperatures combined with ongoing browning of lake waters will promote the most pronounced internal warming in small, shallow, and relatively clear water lakes, that is, the most abundant lake type in northern Sweden (see in Bergström & Karlsson, 2019). Our results support recent research advances pointing out that warming of surface waters and consequent cooling of bottom waters of stratified lakes is related to altitude, latitude, and browning, and is stronger in lakes with smaller surface area (Bartosiewicz et al., 2019; Ficker et al., 2017; Pilla et al., 2018, 2020). However, our results suggest that instead of lake area, average lake depth (z_avg_) predicts internal warming best, and that even among shallow lakes (up to 16 m depth in our dataset) lake bathymetry impacts internal warming. In the context of ongoing global change, internal warming of lake surface waters is therefore likely to continue to increase not only in Sweden, but also on a global scale (IPCC, 2021; O'Reilly et al., 2015). At northern latitudes where small and shallow lakes are globally most abundant (Verpoorter et al., 2014), and warming and lake browning are especially pronounced (Creed et al., 2018; Pagano et al., 2014; Roulet & Moore, 2006; Solomon et al., 2015), internal warming of lake surface waters is likely to accelerate and thus affect pelagic GPP.

### 4.2 Pelagic GPP in lakes over the northern Swedish landscape

**FIGURE 7:**
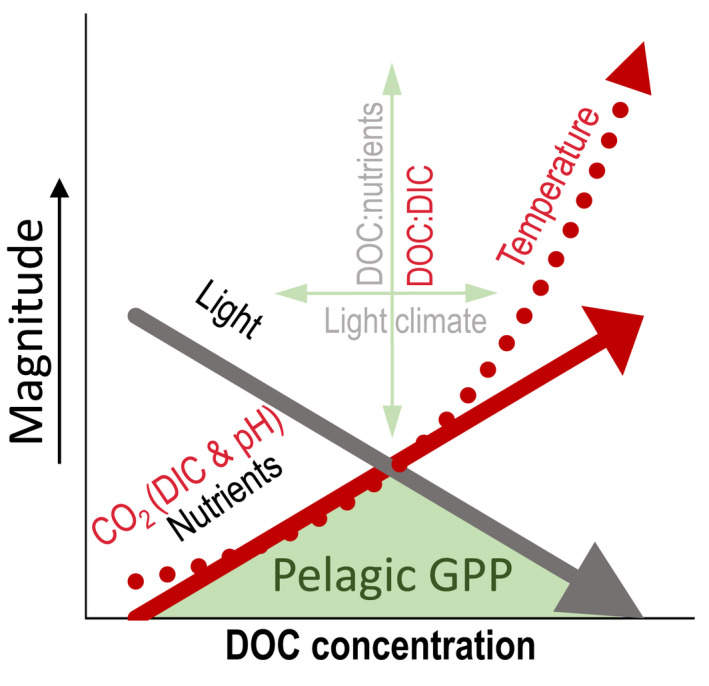
Conceptual overview of how our results (text and arrows in red) add to current knowledge (text and arrows in black/gray) regarding drivers of summer pelagic GPP (green) with increasing DOC. Consensus is that GPP is initially limited by nutrients, and at higher DOC by light inhibition. The height of the peak in GPP is defined by the DOC:nutrient ratio, and the location of the GPP peak by the coloring of DOC (light climate). Our results point out additional major drivers of summer pelagic GPP, which are CO_2_ concentrations (determined by lake pH and the total DIC pool) increasing with DOC that together with DOC:DIC stoichiometry further enhance GPP. Increased water temperatures with increasing DOC can promote GPP rates even more and thus partly counteract the negative impact of reduced light conditions caused by DOC on pelagic GPP.

Interestingly, we found that summer pelagic GPP_z,max_ rates foremost were associated with CO_2_ concentrations (Figures 3, 4, 5), and secondly with water temperature (T_depth_; Figure 3). Moreover, in the PLS regression DOC was selected as second driver, and GPP_z,max_ rates increased with DOC on the first component (positive loading) but decreased on the second component (negative loading), both when including all lakes and lakes in the subarctic and Arctic biome specifically (Figure 4; Table S4). Our results thus confirm previous studies that DOC clusters together with nutrients (TN) and warmer temperatures on the one hand (positive effect on GPP), and reduced light availability on the other hand (negative effect on GPP) (Figures 1a and 7; Table S5; Bergström & Karlsson, 2019; Isles et al., 2021; Kelly et al., 2018; Seekell et al., 2015). Hence, when and how nutrient and light conditions relative to CO_2_ availability and temperature conditions control pelagic GPP rates is a delicate balance and includes interactive effects. In our dataset, summer GPP_z,max_ rates were mostly related to lake CO_2_ concentrations, indicating carbon fertilization. Regardless, although measured in the season and depth with optimal light and temperature conditions, the GPP_z,max_ rates were still impacted by both the dampening (light) and enhancing (nutrients, temperature) effects related with DOC.

In addition, the integrated effect of various drivers on the production (GPP_lake‐average_) showed the strongest relation with CO_2_ and secondly with T_air_ (Figures 3, 4 and 5a). An additional part of the variance in GPP_lake‐average_ decreased with DOC:DIC, and increased with DOC:TP and TP, supporting that the peak in GPP_lake‐average_ was moderated both by DOC:nutrient and DOC:DIC stoichiometry (Table 1 and Figure 1a). Hence, or results suggest co‐fertilization by CO_2_ and nutrients on pelagic GPP, similar to results of previous field studies in oligotrophic northern lakes (Jansson et al., 2012; Vogt et al., 2017). There are several possible explanations and mechanisms behind independent co‐fertilization of CO_2_ and nutrients on pelagic GPP—for example, enzymes active in photosynthesis may not be fully saturated at CO_2_ levels close to equilibrium and/or that the carbon concentrating mechanism of phytoplankton cells is downregulated under nutrient limited conditions (see Jansson et al., 2012 and references therein). Independent co‐fertilization of CO_2_ and nutrients on GPP is also shown in several experimental settings (Hamdan et al., 2018; Low‐Décarie et al., 2011, 2014).

Our observation, showing that the peak of the unimodal distribution of pelagic GPP_lake‐average_ with increasing light attenuation (kd) is influenced by both DOC:nutrient and DOC:DIC stoichiometry (Figure 6a,b) supports the idea that significant changes in pelagic GPP_lake‐average_ due to increased DIC (CO_2_) and nutrient availability only occur when there is sufficient light throughout the water column to promote pelagic GPP. Higher DOC:TP ratios are generally related to lower GPP_lake‐average_ (Bergström & Karlsson, 2019; Isles et al., 2021; Kelly et al., 2018), which is opposite to our results in the MLR. In our dataset, DOC:DIC explained variation in the peak of GPP_lake‐average_ better than DOC:TP (or the DOC:TN). Possibly, GPP_lake‐average_ was less related to DOC:nutrient compared to DOC:DIC due to different phytoplankton nutrient limitation regimes with more P to NP co‐limited conditions in the subarctic and Arctic lakes, and strict N‐limited conditions in the boreal lakes (Bergström et al., 2013, 2020; Isles et al., 2020 and Figure 4). Interestingly, the GPP_lake‐average_ showed a clear peak at water temperatures around 20°C, and the response of GPP_lake‐average_ per DOC to DIC, TP, and TN categories followed the temperature within this interval (Figure 6c,d; Figure S3b), indicating that temperature influences the response in GPP to changes in limiting inorganic carbon and limiting nutrient concentrations. Interactive effects of nutrients and temperature on pelagic GPP are well‐known (Björk‐Ramberg & Ånell, 1985; Faithfull et al., 2011; Lewis, 2011), but have not been considered in relation to DOC:nutrient, or DOC:DIC stoichiometry. Altogether, our results imply that lake inorganic carbon (CO_2_, DIC) availability and temperature are additional drivers of summer pelagic GPP (rates and lake‐averages), besides light and nutrients conditions governed by DOC, in lakes across the northern Swedish landscape.

### 4.3 Pelagic GPP per biome

We found slightly different drivers for GPP among biomes, likely as an effect of biome differences in climate, catchment properties, and atmospheric N deposition (Elser et al., 2009; Lewis, 2011). For the boreal biome, lakes spanned a wide gradient in DOC concentrations, and were overall browner, warmer, and richer in nutrients, with more variable pelagic GPP (rates and lake‐averages) that was mainly related to CO_2_ and DOC:DIC (Figure 4b,f; Table S4). The subarctic lakes had less variable lake water temperature, DOC, and nutrient concentrations, and intermediate pelagic GPP being mostly related to temperature (Figure 4c,g; Table S4). The Arctic lakes were generally of low DOC, clear, cold, nutrient poor, and some not stratified, and generally low pelagic GPP_z,max_ rates mostly associated with DIC and TP (Figure 4d,h; Table S4). The observed low pelagic GPP in the Arctic lakes (especially at high altitudes) is suggested to be an effect of low water temperatures leading to low growth rates even during conditions of nutrient enrichment (Bergström et al., 2013). Yet, as temperatures and nutrients are all low, relatively small changes in environmental conditions are likely to promote shifts from one limiting factor to another. Furthermore, the differences in environmental drivers of pelagic GPP that we identified for the northern Swedish landscape, and for the different biomes, emphasize the importance of including different biomes when upscaling to understand climate change effects on larger scales.

### 4.4 Inorganic carbon

Altogether, our results underline the importance of DIC and CO_2_ for pelagic GPP in addition to nutrients, light and temperature conditions, and bathymetry. While recent studies have shown positive effects of DIC (and CO_2_) on pelagic GPP (Hammer, 2019; Kragh & Sand‐Jensen, 2018), there are still quite few studies where DIC effects on pelagic GPP are studied empirically (Hessen et al., 2017; Jansson et al., 2012; Vogt et al., 2017). The fraction of CO_2_ in the DIC pool is a function of pH, slightly moderated by temperature (see Section 2.3), and is relevant for GPP since the (bi)carbonate part of DIC is generally not as favorable for phytoplankton growth (with the exception for cyanobacteria) as CO_2_ (Huisman et al., 2018; Wetzel, 2001). We included both DIC and CO_2_ in the models for explaining summer pelagic GPP_z,max_ rates and GPP_lake‐averages,_ which were both strongly related to CO_2_ (explaining 56% and 53% of the variance in GPP in the MLR, respectively), and GPP_lake‐averages_ additionally decreased with increasing DOC:DIC stoichiometry. In the Arctic, however, where lakes were more alkaline (higher pH) and the fraction of CO_2_ was lower (Figure 5b), DIC (and not CO_2_) was selected as a major driver (Table S1). Hence, since the contribution of CO_2_ to total DIC can be highly variable when assessing lakes in different landscapes with different catchment properties (Figure 5b), DIC might be a poor proxy for CO_2_ availability.

CO_2_ supersaturation is ubiquitous throughout lakes at higher latitudes caused by high input of CO_2_ and organic material from land (Åberg et al., 2010; Sobek et al., 2003). However, natural variability of pH across landscapes related to catchment characteristics and in situ metabolism and biological engineering (Huisman et al., 2018; Paerl et al., 2016; Verspagen et al., 2014), but also anthropogenic influences such as increasing atmospheric CO_2_ concentrations and ongoing recovery from acidification (Garmo et al., 2014; IPCC, 2021; Isles et al., 2018; Skjelkvåle et al., 2001) are likely to impact the amounts and form of inorganic carbon available for photosynthesis. Enhanced inorganic carbon availability in the form of CO_2_ will promote summer pelagic GPP according to our results, but could also potentially impact phytoplankton community composition, favoring species that lack the ability to use (bi)carbonate as an inorganic carbon source (e.g., chrysophytes; Bhatti & Colman, 2005; Maberly et al., 2009), relative to species that have the ability to do so (e.g., cyanobacteria; Huisman et al., 2018; Verspagen et al., 2014). Enhanced pH accompanied with lake warming, may thus further promote cyanobacteria over other species of phytoplankton (Huisman et al., 2018; Verspagen et al., 2014).

### 4.5 Drivers of pelagic productivity in lakes across the northern landscape

Our results, together with others, can be applied to identify potential trajectories for pelagic GPP in northern latitude lakes following global change. Adding to previous studies recognizing the importance of DOC, and DOC:nutrient stoichiometry, impacting light and nutrient availability (Bergström & Karlsson, 2019; Kelly et al., 2018; Isles et al., 2021; Rivera Vasconcelos et al., 2018), our study further emphasizes the role of inorganic carbon, DOC:DIC stoichiometry, and temperature for pelagic GPP in summer (summarized in Figure 7). Our results imply that for northern Sweden global warming combined with browning likely enhances summer pelagic GPP via effects on water temperature, nutrients, and CO_2_. Considering the large‐scale impacts and similarities of global warming and browning on lakes at higher latitudes throughout the northern hemisphere (Cohen et al., 2014; Creed et al., 2018; Solomon et al., 2015), these recognized changes are likely to operate on a global scale.

However, our results also emphasize that caution should be made when upscaling and using space‐for‐time substitutions. We use summer data from different years representing only a snapshot in time, where the lake environmental conditions and subsequent responses in GPP across northern Swedish landscapes have been influenced by atmospheric acid deposition and differences in catchment vegetation cover (see Isles et al., 2018, 2020). Yet, when analyzing air temperature data for all years within the 20‐year sampling period, apparent differences in climate (air temperature) are consistently greater among than within biomes (Figure S1). The sampling occasions were within the natural variation in each biome, indicating that the temperature effect observed in our results is unrelated to individual sampling years. Thus, here a space‐for‐time approach is valid for assessing key environmental drivers of summer pelagic GPP across the northern Swedish landscape. However, more research is required, and consideration needs to be taken on to what extent, for example, the internal warming of lakes is moderated by DOC aromaticity and lake size (area, depth), and how biome‐specific differences and seasonality might influence the importance of different environmental drivers on pelagic GPP when upscaling.

## CONFLICT OF INTEREST

Authors declare no conflict of interest.

## Supporting information


**Appendix S1:** Supporting Information

## Data Availability

The data that support the findings of this study are openly available in https://doi.org/10.5061/dryad.t1g1jwt5k.

## References

[gcb16469-bib-0001] Åberg, J. , Jansson, M. , & Jonsson, A. (2010). Importance of water temperature and thermal stratification dynamics for temporal variation of surface water CO_2_ in a boreal lake. Journal of Geophysical Research: Biogeosciences, 115(G2), 549–560. 10.1029/2009jg001085

[gcb16469-bib-0002] AMAP . (1998). AMAP assessment report. In AMAP assessment report: Arctic pollution issues (pp. 9–24). ISBN 82‐7655‐061‐4. https://www.amap.no/documents/doc/amap‐assessment‐report‐arctic‐pollution‐issues/68

[gcb16469-bib-0003] Ask, J. , Karlsson, J. , Persson, L. , Ask, P. , Byström, P. , & Jansson, M. (2009). Terrestrial organic matter and light penetration: Effects on bacterial and primary production in lakes. Limnology and Oceanography, 54(6), 2034–2040. 10.4319/lo.2009.54.6.2034

[gcb16469-bib-0004] Bartosiewicz, M. I. , Laurion, I. , Clayer, F. , & Maranger, R. (2016). Heat‐wave effects on oxygen, nutrients, and phytoplankton can alter global warming potential of gases emitted from a small shallow lake. Environmental Science & Technology, 50(12), 6267–6275. 10.1021/acs.est.5b06312 27266257

[gcb16469-bib-0005] Bartosiewicz, M. , Przytulska, A. , Lapierre, J. , Laurion, I. , Lehmann, M. F. , & Maranger, R. (2019). Hot tops, cold bottoms: Synergistic climate warming and shielding effects increase carbon burial in lakes. Limnology and Oceanography Letters, 4(5), 132–144. 10.1002/lol2.10117

[gcb16469-bib-0006] Bergström, A. K. , Faithfull, C. , Karlsson, D. , & Karlsson, J. (2013). Nitrogen deposition and warming—effects on phytoplankton nutrient limitation in subarctic lakes. Global Change Biology, 19, 2557–2568. 10.1111/gcb.12234 23629960

[gcb16469-bib-0007] Bergström, A. K. , Jonsson, A. , Isles, P. D. F. , Creed, I. F. , & Lau, D. C. P. (2020). Changes in nutritional quality and nutrient limitation regimes of phytoplankton in response to declining N deposition in mountain lakes. Aquatic Sciences, 82(2), 1–16. 10.1007/s00027-020-0697-1 32489242

[gcb16469-bib-0008] Bergström, A. K. , & Karlsson, J. (2019). Light and nutrient control phytoplankton biomass responses to global change in northern lakes. Global Change Biology, 25(6), 2021–2029. 10.1111/gcb.14623 30897262

[gcb16469-bib-0009] Bhatti, S. , & Colman, B. (2005). Inorganic carbon acquisition by the chrysophyte alga Mallomonas papillosa Bhatti and Colman. Canadian Journal of Botany, 83(7), 891–897. 10.1139/b05-075

[gcb16469-bib-0010] Björk‐Ramberg, S. , & Ånell, C. (1985). Production and chlorophyll concentration of epipelic and epilithic algae in fertilized and nonfertilized subarctic lakes. Hydrobiologia, 126(3), 213–219. 10.1007/BF00007498

[gcb16469-bib-0011] Brown, T. W. , Lajeunesse, M. J. , & Scott, K. M. (2019). Strong effects of elevated CO_2_ on freshwater microalgae and ecosystem chemistry. Limnology and Oceanography, 65(2), 304–313. 10.1002/lno.11298

[gcb16469-bib-0012] Carpenter, S. R. , Cole, J. J. , Kitchell, J. F. , & Pace, M. L. (1998). Impact of dissolved organic carbon, phosphorus, and grazing on phytoplankton biomass and production in experimental lakes. Limnology and Oceanography, 43(1), 73–80. 10.4319/lo.1998.43.1.0073

[gcb16469-bib-0013] Cohen, J. , Screen, J. A. , Furtado, J. C. , Barlow, M. , Whittleston, D. , Coumou, D. , Francis, J. , Dethloff, K. , Entekhabi, D. , Overland, J. , & Jones, J. (2014). Recent Arctic amplification and extreme mid‐latitude weather. Nature Geoscience, 7(9), 627–637. 10.1038/ngeo2234

[gcb16469-bib-0014] Creed, I. F. , Bergström, A.‐K. , Trick, C. G. , Grimm, N. B. , Hessen, D. O. , Karlsson, J. , Kidd, K. A. , Kritzberg, E. , McKnight, D. M. , Freeman, E. C. , Senar, O. E. , Andersson, A. , Ask, J. , Berggren, M. , Cherif, M. , Giesler, R. , Hotchkiss, E. R. , Kortelainen, P. , Palta, M. M. , … Weyhenmeyer, G. A. (2018). Global change‐driven effects on dissolved organic matter composition: Implications for food webs of northern lakes. Global Change Biology, 24(8), 3692–3714. 10.1111/gcb.14129 29543363

[gcb16469-bib-0015] De Wit, H. A. , Valinia, S. , Weyhenmeyer, G. A. , Futter, M. N. , Kortelainen, P. , Austnes, K. , Hessen, D. O. , Räike, A. , Laudon, H. , & Vuorenmaa, J. (2016). Current browning of surface waters will be further promoted by wetter climate. Environmental Science and Technology Letters, 3(12), 430–435. 10.1021/acs.estlett.6b00396

[gcb16469-bib-0016] Deininger, A. , Faithfull, C. L. , Karlsson, J. , Klaus, M. , & Bergström, A. K. (2017). Pelagic food web response to whole lake N fertilization. Limnology and Oceanography, 62(4), 1498–1511. 10.1002/lno.10513

[gcb16469-bib-0017] Deininger, A. , Jonsson, A. , Karlsson, J. , & Bergström, A. K. (2019). Pelagic food webs of humic lakes show low short‐term response to forest harvesting. Ecological Applications, 29(1), 1–13. 10.1002/eap.1813 30312509

[gcb16469-bib-0018] Del Giorgio, P. A. , Cole, J. J. , Caraco, N. F. , & Peters, R. H. (1999). Linking planktonic biomass and metabolism to net gas fluxes in northern temperate lakes. Ecology, 80(4), 1422–1431. 10.1890/0012-9658(1999)080[1422:LPBAMT]2.0.CO;2

[gcb16469-bib-0019] Elser, J. J. , Andersen, T. , Baron, J. S. , Bergström, A. K. , Jansson, M. , Kyle, M. , Nydick, K. R. , Steger, L. , & Hessen, D. O. (2009). Shifts in lake N: P stoichiometry and nutrient limitation driven by atmospheric nitrogen deposition. Science, 326, 835–837. 10.1126/science.1176199 19892979

[gcb16469-bib-0020] Faithfull, C. L. , Bergström, A. K. , & Vrede, T. (2011). Effects of nutrients and physical lake characteristics on bacterial and phytoplankton production: A meta‐analysis. Limnology and Oceanography, 56(5), 1703–1713. 10.4319/lo.2011.56.5.1703

[gcb16469-bib-0021] Ficker, H. , Luger, M. , & Gassner, H. (2017). From dimictic to monomictic: Empirical evidence of thermal regime transitions in three deep alpine lakes in Austria induced by climate change. Freshwater Biology, 62(8), 1335–1345. 10.1111/fwb.12946

[gcb16469-bib-0022] Finstad, A. G. , Andersen, T. , Larsen, S. , Tominaga, K. , Blumentrath, S. , De Wit, H. A. , Tømmervik, H. , & Hessen, D. O. (2016). From greening to browning: Catchment vegetation development and reduced S‐deposition promote organic carbon load on decadal time scales in Nordic lakes. Scientific Reports, 6(7485), 1–8. 10.1038/srep31944 27554453 PMC4995398

[gcb16469-bib-0023] Garmo, Ø. A. , Skjelkvåle, B. L. , De Wit, H. A. , Colombo, L. , Curtis, C. , Fölster, J. , Hoffmann, A. , Hruška, J. , Høgåsen, T. , Jeffries, D. S. , Keller, W. B. , Krám, P. , Majer, V. , Monteith, D. T. , Paterson, A. M. , Rogora, M. , Rzychon, D. , Steingruber, S. , John, L. , … Worsztynowicz, A. (2014). Trends in surface water chemistry in acidified areas in Europe and North America from 1990 to 2008. Water, Air, & Soil Pollution, 225(3). 10.1007/s11270-014-1880-6

[gcb16469-bib-0024] Graham, G. , Cook, W. , Graham, L. E. , Graham, J. M. , Wilcox, L. W. , & Cook, M. E. (n.d.). Graham | Graham | Wilcox | Cook. ISBN 978‐0‐9863935‐4‐9.

[gcb16469-bib-0025] Hamdan, M. , Byström, P. , Hotchkiss, E. R. , Al‐Haidarey, M. J. , Ask, J. , & Karlsson, J. (2018). Carbon dioxide stimulates lake primary production. Scientific Reports, 8, 10878. 10.1038/s41598-018-29166-3 30022034 PMC6052161

[gcb16469-bib-0026] Hammer, K. J. (2019). Inorganic carbon promotes photosynthesis, growth, and maximum biomass of phytoplankton in eutrophic water bodies. Freshwater Biology, 64, 1956–1970. 10.1111/fwb.13385

[gcb16469-bib-0027] Hansen, J. , Ruedy, R. , Sato, M. , & Lo, K. (2010). Global surface temperature change. Reviews of Geophysics, 48, RG4004. 10.1029/2010RG000345

[gcb16469-bib-0028] Hessen, D. O. , Håll, J. P. , Thrane, J. E. , & Andersen, T. (2017). Coupling dissolved organic carbon, CO_2_ and productivity in boreal lakes. Freshwater Biology, 62(5), 945–953. 10.1111/fwb.12914

[gcb16469-bib-0029] Houser, J. N. (2006). Water color affects the stratification, surface temperature, heat content, and mean epilimnetic irradiance of small lakes. Canadian Journal of Fisheries and Aquatic Sciences, 63(11), 2447–2455. 10.1139/f06-131

[gcb16469-bib-0030] Hudson, J. J. , Dillon, P. J. , & Somers, K. M. (2003). Long‐term patterns in dissolved organic carbon in boreal lakes: The role of incident radiation, precipitation, air temperature, southern oscillation and acid deposition. Hydrology and Earth System Sciences, 7(3), 390–398. 10.5194/hess-7-390-2003

[gcb16469-bib-0031] Huisman, J. , Codd, G. A. , Paerl, H. W. , Ibelings, B. W. , Verspagen, J. M. H. , & Visser, P. M. (2018). Cyanobacterial blooms. Nature Reviews Microbiology, 16(August), 471–483. 10.1038/s41579-018-0040-1 29946124

[gcb16469-bib-0032] IPCC (2021). Summary for policymakers. In V. Masson‐Delmotte , P. Zhai , A. Pirani , S. L. Connors , C. Péan , S. Berger , N. Caud , Y. Chen , L. Goldfarb , M. I. Gomis , M. Huang , K. Leitzell , E. Lonnoy , J. B. R. Matthews , T. K. Maycock , T. Waterfield , O. Yelekçi , R. Yu , & B. Zhou (Eds.), Climate change 2021: The physical science basis. Contribution of working group I to the sixth assessment report of the Intergovernmental Panel on Climate Change. Cambridge University Press In Press.

[gcb16469-bib-0033] Isles, P. D. F. (2020). The misuse of ratios in ecological stoichiometry. Ecology, 101(11), 1–7. 10.1002/ecy.3153 32731303

[gcb16469-bib-0034] Isles, P. D. F. , Creed, I. F. , & Bergström, A. K. (2018). Recent synchronous declines in DIN:TP in Swedish lakes. Global Biogeochemical Cycles, 32(2), 208–225. 10.1002/2017GB005722

[gcb16469-bib-0035] Isles, P. D. F. , Creed, I. F. , Jonsson, A. , & Bergström, A.‐K. (2021). Trade‐offs between light and nutrient availability across gradients of dissolved organic carbon lead to spatially and temporally variable responses of lake phytoplankton biomass to browning. Ecosystems, 24, 1837–1852. 10.1007/s10021-021-00619-7

[gcb16469-bib-0036] Isles, P. D. F. , Jonsson, A. , Creed, I. F. , & Bergström, A. K. (2020). Does browning affect the identity of limiting nutrients in lakes? Aquatic Sciences, 82(2), 1–14. 10.1007/s00027-020-00718-y 32489242

[gcb16469-bib-0037] Jansson, M. , Karlsson, J. , & Jonsson, A. (2012). Carbon dioxide supersaturation promotes primary production in lakes. Ecology Letters, 15(6), 527–532. 10.1111/j.1461-0248.2012.01762.x 22420750

[gcb16469-bib-0038] Jones, R. I. (1992). The influence of humic substances on lacustrine planktonic food chains BT (Chapter 6). In K. Salonen , T. Kairesalo , & R. I. Jones (Eds.), Dissolved organic matter in lacustrine ecosystems (pp. 73–91). Kluwer Academic Publisher. http://www.springerlink.com/index/10.1007/978‐94‐011‐2474‐4_6%5Cnpapers3://publication/doi/10.1007/978‐94‐011‐2474‐4_6

[gcb16469-bib-0039] Jones, R. I. (1998). Phytoplankton, primary production and nutrient cycling. In D. O. Hessen & L. J. Tranvik (Eds.), Aquatic humic substances: Ecology and biogeochemistry (pp. 145–175). Springer Berlin Heidelberg. 10.1007/978-3-662-03736-2_8

[gcb16469-bib-0040] Jonsson, A. , Meili, M. , Bergström, A. K. , & Jansson, M. (2001). Whole‐lake mineralization of allochthonous and autochthonous organic carbon in a large humic lake (Örträsket, N. Sweden). Limnology and Oceanography, 46(7), 1691–1700. 10.4319/lo.2001.46.7.1691

[gcb16469-bib-0041] Karlsson, J. , Jonsson, A. , & Jansson, M. (2001). Bacterioplankton production in lakes along an altitude gradient in the subarctic north of Sweden. Microbial Ecology, 42(3), 372–382. 10.1111/j.1365-2427.2007.01725.x 12024262

[gcb16469-bib-0042] Karlsson, J. , Jonsson, A. , & Jansson, M. (2005). Productivity of high‐latitude lakes: Climate effect inferred from altitude gradient. Global Change Biology, 11(5), 710–715. 10.1111/j.1365-2486.2005.00945.x

[gcb16469-bib-0043] Kelly, P. T. , Solomon, C. T. , Zwart, J. A. , & Jones, S. E. (2018). A framework for understanding variation in pelagic gross primary production of lake ecosystems. Ecosystems, 21(7), 1364–1376. 10.1007/s10021-018-0226-4

[gcb16469-bib-0044] Klug, J. L. (2002). Positive and negative effects of allochthonous dissolved organic matter and inorganic nutrients on phytoplankton growth. Canadian Journal of Fisheries and Aquatic Sciences, 59(1), 85–95. 10.1139/f01-194

[gcb16469-bib-0045] Klug, J. L. (2005). Bacterial response to dissolved organic matter affects resource availability for algae. Canadian Journal of Fisheries and Aquatic Sciences, 62(2), 472–481. 10.1139/f04-229

[gcb16469-bib-0046] Kragh, T. , & Sand‐Jensen, K. (2018). Carbon limitation of lake productivity. Proceedings of the Royal Society B: Biological Sciences, 285(1891), 20181415. 10.1098/rspb.2018.1415 PMC6253374 30429299

[gcb16469-bib-0047] Kritzberg, E. S. (2017). Centennial‐long trends of lake browning show major effect of afforestation. Limnology and Oceanography Letters, 2(4), 105–112. 10.1002/lol2.10041

[gcb16469-bib-0048] Larsen, S. , Andersen, T. , & Hessen, D. O. (2011). The pCO_2_ in boreal lakes: Organic carbon as a universal predictor? Global Biogeochemical Cycles, 25(2), 1–8. 10.1029/2010GB003864

[gcb16469-bib-0049] Laudon, H. , Buttle, J. , Carey, S. K. , McDonnell, J. , McGuire, K. , Seibert, J. , Shanley, J. , Soulsby, C. , & Tetzlaff, D. (2012). Cross‐regional prediction of long‐term trajectory of stream water DOC response to climate change. Geophysical Research Letters, 39(17), 4–9. 10.1029/2012GL053033

[gcb16469-bib-0050] Laudon, H. , Hasselquist, E. M. , Peichl, M. , Lindgren, K. , Sponseller, R. , Lidman, F. , Kuglerová, L. , Hasselquist, N. J. , Bishop, K. , Nilsson, M. B. , & Ågren, A. M. (2021). Northern landscapes in transition: Evidence, approach and ways forward using the Krycklan Catchment Study. Hydrological Processes, 35(4), 1–15. 10.1002/hyp.14170

[gcb16469-bib-0051] Lazzarino, J. K. , Bachmann, R. W. , Hoyer, M. V. , & Canfield, D. E. (2009). Carbon dioxide supersaturation in Florida lakes. Hydrobiologia, 627(1), 169–180. 10.1007/s10750-009-9723-y

[gcb16469-bib-0052] Lehner, B. , & Döll, P. (2004). Development and validation of a global database of lakes, reservoirs and wetlands. Journal of Hydrology, 296(1–4), 1–22. 10.1016/j.jhydrol.2004.03.028

[gcb16469-bib-0053] Lewis, W. M., Jr . (2011). Global primary production of lakes: 19 th Baldi Memorial Lecture, Inland Waters, 1(1), 1–28. 10.5268/IW-1.1.384

[gcb16469-bib-0054] Lind, P. , & Kjellström, E. (2008). Temperature and precipitation changes in Sweden; a wide range of model‐based projections for the 21st century. SMHI, number RMK 113. https://www.smhi.se/en/publications/temperature‐and‐precipitation‐changes‐in‐sweden‐a‐wide‐range‐of‐model‐based‐projections‐for‐the‐21st‐century‐1.6647

[gcb16469-bib-0055] Low‐Décarie, E. , Fussmann, G. F. , & Bell, G. (2011). The effect of elevated CO_2_ on growth and competition in experimental phytoplankton communities. Global Change Biology, 17(8), 2525–2535. 10.1111/j.1365-2486.2011.02402.x

[gcb16469-bib-0056] Low‐Décarie, E. , Fussmann, G. F. , & Bell, G. (2014). Aquatic primary production in a high‐CO_2_ world. Trends in Ecology & Evolution, 29(4), 223–232. 10.1016/j.tree.2014.02.006 24631287

[gcb16469-bib-0057] Maberly, S. C. , Ball, L. A. , Raven, J. A. , & Sültemeyer, D. (2009). Inorganic carbon acquisition by chrysophytes. Journal of Phycology, 45(5), 1052–1061. 10.1111/j.1529-8817.2009.00734.x 27032350

[gcb16469-bib-0058] Mehmood, T. , Liland, K. H. , Snipen, L. , & Sæbø, S. (2012). A review of variable selection methods in partial least squares regression. Chemometrics and Intelligent Laboratory Systems, 118, 62–69. 10.1016/j.chemolab.2012.07.010

[gcb16469-bib-0059] Mevik, B. H. , & Wehrens, R. (2007). The pls package: Principal component and partial least squares regression in R. Journal of Statistical Software, 18(2), 1–23. 10.18637/jss.v018.i02

[gcb16469-bib-0060] Müller‐Navarra, D. C. (2008). Food web paradigms: The biochemical view on trophic interactions. International Review of Hydrobiology, 93(4–5), 489–505. 10.1002/iroh.200711046

[gcb16469-bib-0061] Nydahl, A. C. , Wallin, M. B. , & Weyhenmeyer, G. A. (2020). Diverse drivers of long‐term pCO_2_ increases across thirteen boreal lakes and streams. Inland Waters, 10(3), 360–372. 10.1080/20442041.2020.1740549

[gcb16469-bib-0062] O'Reilly, C. M. , Rowley, R. J. , Schneider, P. , Lenters, J. D. , Mcintyre, P. B. , Kraemer, B. M. , Weyhenmeyer, G. A. , Straile, D. , Dong, B. , Adrian, R. , Allan, M. G. , Anneville, O. , Arvola, L. , Austin, J. , Bailey, J. L. , Baron, J. S. , Brookes, J. D. , de Eyto, E. , Dokulil, M. T. , … Zhang, G. (2015). Rapid and highly variable warming of lake surface waters around the globe. Geophysical Research Letters, 1–9. 10.1002/2015GL066235.Received

[gcb16469-bib-0063] Paerl, H. W. , Otten, T. G. , & Alan, R. (2016). Moving towards adaptive management of cyanotoxin‐impaired water bodies. Microbial Biotechnology, 9, 641–651. 10.1111/1751-7915.12383 27418325 PMC4993183

[gcb16469-bib-0064] Pagano, T. , Bida, M. , & Kenny, J. (2014). Trends in levels of allochthonous dissolved organic carbon in natural water: A review of potential mechanisms under a changing climate. Water, 6(10), 2862–2897. 10.3390/w6102862

[gcb16469-bib-0065] Peltomaa, E. T. , Aalto, S. L. , Vuorio, K. M. , & Taipale, S. J. (2017). The importance of phytoplankton biomolecule availability for secondary production. Frontiers in Ecology and Evolution, 5(October), 1–12. 10.3389/fevo.2017.00128

[gcb16469-bib-0066] Pilla, R. M. , Williamson, C. E. , Adamovich, B. V. , Adrian, R. , Anneville, O. , Chandra, S. , Colom‐Montero, W. , Devlin, S. P. , Dix, M. A. , Dokulil, M. T. , Gaiser, E. E. , Girdner, S. F. , Hambright, K. D. , Hamilton, D. P. , Havens, K. , Hessen, D. O. , Higgins, S. N. , Huttula, T. H. , Huuskonen, H. , … Zadereev, E. (2020). Deeper waters are changing less consistently than surface waters in a global analysis of 102 lakes. Scientific Reports, 10, 20514. 10.1038/s41598-020-76873-x 33239702 PMC7688658

[gcb16469-bib-0067] Pilla, R. M. , Williamson, C. E. , Zhang, J. , Smyth, R. L. , Lenters, J. D. , Brentrup, J. A. , Knoll, L. B. , & Fisher, T. J. (2018). Browning‐related decreases in water transparency lead to long‐term increases in surface water temperature and thermal stratification in two small lakes. Journal of Geophysical Research: Biogeosciences, 123(5), 1651–1665. 10.1029/2017JG004321

[gcb16469-bib-0068] Puts, I. C. , Bergström, A.‐K. , Verheijen, H. A. , Norman, S. , & Ask, J. (2022). An ecological and methodological assessment of benthic gross primary production in northern lakes. Ecosphere, 13(3), 1–16. 10.1002/ecs2.3973

[gcb16469-bib-0069] Rivera Vasconcelos, F. , Diehl, S. , Rodríguez, P. , Karlsson, J. , & Byström, P. (2018). Effects of terrestrial organic matter on aquatic primary production as mediated by pelagic–benthic resource fluxes. Ecosystems, 21(6), 1255–1268. 10.1007/s10021-017-0217-x

[gcb16469-bib-0070] Roulet, N. , & Moore, T. R. (2006). Environmental chemistry: Browning the waters. Nature, 444(7117), 283–284. 10.1038/444283a 17108948

[gcb16469-bib-0071] Schindler, D. W. , Schmidt, R. V. , & Reid, R. A. (1972). Acidification and bubbling as an alternative to filtration in determining phytoplankton production by the 14 C method. Journal of the Fisheries Research Board of Canada, 29(11), 1627–1631. 10.1139/f72-250

[gcb16469-bib-0072] Schneider, P. , Hook, S. J. , Radocinski, R. G. , Corlett, G. K. , Hulley, G. C. , Schladow, S. G. , & Steissberg, T. E. (2009). Satellite observations indicate rapid warming trend for lakes in California and Nevada. Geophysical Research Letters, 36(22), 1–6. 10.1029/2009GL040846

[gcb16469-bib-0073] Searle, S. R. , Speed, F. M. , & Milliken, G. A. (1980). Population marginal means in the linear model: an alternative to least squares means. The American Statistician, 34(4), 216–221. 10.1080/00031305.1980.10483031

[gcb16469-bib-0074] Seekell, D. A. , Lapierre, J.‐F. , Ask, J. , Bergström, A.‐K. , Deininger, A. , Rodríguez, P. , & Karlsson, J. (2015). The influence of dissolved organic carbon on primary production in northern lakes. Limnology and Oceanography, 60(4), 1276–1285. 10.1002/lno.10096

[gcb16469-bib-0075] Serreze, M. C. , Barrett, A. P. , Stroeve, J. C. , Kindig, D. N. , & Holland, M. M. (2009). The emergence of surface‐based Arctic amplification. The Cryosphere, 3(1), 11–19. 10.5194/tc-3-11-2009

[gcb16469-bib-0076] Skjelkvåle, B. L. , Mannio, J. , Wilander, A. , & Andersen, T. (2001). Recovery from acidification of lakes in Finland, Norway and Sweden 1990–1999. Hydrology and Earth System Sciences, 5(3), 327–338. 10.5194/hess-5-327-2001

[gcb16469-bib-0077] Sobek, S. , Algesten, G. , Bergström, A. K. , Jansson, M. , & Tranvik, L. J. (2003). The catchment and climate regulation of pCO_2_ in boreal lakes. Global Change Biology, 9(4), 630–641. 10.1046/j.1365-2486.2003.00619.x

[gcb16469-bib-0078] Solomon, C. T. , Jones, S. E. , Weidel, B. C. , Buffam, I. , Fork, M. L. , Karlsson, J. , Larsen, S. , Lennon, J. T. , Read, J. S. , Sadro, S. , & Saros, J. E. (2015). Ecosystem consequences of changing inputs of terrestrial dissolved organic matter to lakes: Current knowledge and future challenges. Ecosystems, 18(3), 376–389. 10.1007/s10021-015-9848-y

[gcb16469-bib-0079] Sterner, R. W. , & Hessen, D. O. (1994). Algal nutrient limitation and the nutrition of aquatic herbivores. Annual Review of Ecology and Systematics, 25, 1–29.

[gcb16469-bib-0080] Stets, E. G. , Butman, D. , McDonald, C. P. , Stackpoole, S. M. , DeGrandpre, M. D. , & Striegl, R. G. (2017). Carbonate buffering and metabolic controls on carbon dioxide in rivers. Global Biogeochemical Cycles, 31(4), 663–677. 10.1002/2016GB005578

[gcb16469-bib-0081] Tetzlaff, D. , Soulsby, C. , Buttle, J. , Capell, R. , Carey, S. K. , Laudon, H. , McDonnell, J. , McGuire, K. , Seibert, J. , & Shanley, J. (2013). Catchments on the cusp? Structural and functional change in northern ecohydrology. Hydrological Processes, 27(5), 766–774. 10.1002/hyp.9700

[gcb16469-bib-0082] Verpoorter, C. , Kutser, T. , Seekell, D. A. , & Tranvik, L. J. (2014). A global inventory of lakes based on high‐resolution satellite imagery. Geophysical Research Letters, 41(18), 6396–6402. 10.1002/2014GL060641

[gcb16469-bib-0083] Verspagen, J. M. H. , Van De Waal, D. B. , Finke, J. F. , Visser, P. M. , Van Donk, E. , & Huisman, J. (2014). Rising CO_2_ levels will intensify phytoplankton blooms in eutrophic and hypertrophic lakes. PLoS One, 9(8), e104325. 10.1371/journal.pone.0104325 25119996 PMC4132121

[gcb16469-bib-0084] Vogt, R. J. , St‐Gelais, N. F. , Bogard, M. J. , Beisner, B. E. , & del Giorgio, P. A. (2017). Surface water CO_2_ concentration influences phytoplankton production but not community composition across boreal lakes. Ecology Letters, 20(11), 1395–1404. 10.1111/ele.12835 29044973

[gcb16469-bib-0085] Wetzel, R. G. (2001). Limnology. In Lake and river ecosystems (3rd ed.). Academic Press.

[gcb16469-bib-0086] Wetzel, R. G. , & Likens, G. E. (1991). Limnological analyses (2nd ed.). Springer‐verlag.

