# Supplementary material for: Browning affects pelagic productivity in northern lakes by surface water warming and carbon fertilization
Source: Glob Chang Biol. 2022 Oct 19;29(2):375–90. doi: 10.1111/gcb.16469 (PMC10092479; doi:10.1111/gcb.16469)
Supplement: Supplementary file 1 — Appendix S1: Supporting Information [file GCB-29-375-s001.docx]

Supporting information. Puts et al., 2022. Browning affects pelagic productivity in northern lakes by surface water warming and carbon fertilization.

Appendix S1

Background data.

Tables

S1 Variables/parameters with their symbols and definition, and units

S2 Lake metadata

S3 ANOVAs water physico chemistry per biome

S4 PLS statistics of (a) GPP_z,max_ rates and (b) GPP_lake-average_

S5 Pearson correlations

Figures

S1 Average monthly temperatures

S2 Kd and DOC

S3 DOC:TN against Kd and temperature

Table S1: Variables/parameters with their symbols and definition, and units.

| **Variable/parameter** | **Symbol** | **Source/equation** | **Unit** |
| --- | --- | --- | --- |
| Depth | z | Direct measurement | m |
| Maximum depth | z_max_ | Bathymetry | m |
| Euphotic depth | z_euph_ | -Ln (0.01)$\cdot$ Kd | m |
| Average lake depth | z_avg_ | V_lake_$\cdot$A_lake_^-1^ | m |
| Lake surface area | A_lake_ | Bathymetry | hectare |
| Lake volume | V_lake_ | Bathymetry | m^3^ |
| Air temperature | T_air_ | Direct measurement | °C |
| Water temperature  (at 0.2m depth) | T_water_ | Direct measurement | °C |
| Internal warming | - | T_water_ - T_air_ | °C |
| % of surface light reaching a discrete depth | %light | 100·e^-kd·z^ | % |
| Volume between depths | V(z) | $\int_{z1}^{z2} V\left( z \right)\cdot dz$ | m^3^ |
| Pelagic GPP rate at discrete depth | GPP*_z_* | Direct measurement | mgC·m^-3^·day^-1^ |
| Maximum pelagic GPP rate found over water column | GPP_z,max_ | Selected measurement | mgC·m^-3^·day^-1^ |
| Pelagic GPP lake average, bathymetry considered | GPP_lake-average_ | $\frac{\int_{0}^{zmax} GPP\left( z \right)\text{·}V\left( z \right)\text{·}dz}{ALake}$ | mg C·m^-2^ ·day^-1^ |

## Table S2: Location, climatic data, water physio-chemistry and bathymetry for our 45 sampled lakes (averages are given per biome in bold). Abbreviations are as follows: DOC= dissolved organic carbon, DIC= dissolved inorganic carbon, TN= total nitrogen, TP= total phosphorus, PAR= daily incoming PAR, Kd= light attenuation coefficient, T_water_= temperature at 0.2m depth, T_air_= monthly average air temperature before sampling, Int.warming= internal warming (T_water_-T_air_), Area= lake surface area, ha.= hectare, z_max_= maximum lake depth, z_avg_= average lake depth, Lljusv. 1S= Ljusvattentjärn 1 South, Lljusv. 2N= Lljusvattentjärn 2 North. Value in bold are not included in averages.

| **Biome**/Lake | **Location** | | |  | **Water physio chemistry** | | | | | |  |  | **Temperature** | | | | **Bathymetry** | | |
| --- | --- | --- | --- | --- | --- | --- | --- | --- | --- | --- | --- | --- | --- | --- | --- | --- | --- | --- | --- |
|  | Lat. | Long. | Altitude | DOC | | DIC | CO_2_ | TN | TP | PAR |  | pH | Kd | T_water_ | T_air_ | Int.  warming | Area | z_max_ | z_avg_ |
|  | Decimal degrees | | m.a.s.l. | mg·L^-1^ | | |  | µg·L^-1^ | | W·m^-^² |  |  | m^-1^ | °C | °C | °C | ha. | m | m |
| **Boreal** |  |  | **283** | **11.1** | | **1.5** | **0.89** | **338** | **13.0** | **7745** |  | **6.3** | **2.1** | **19.3** | **14.6** | **4.9** | **3.4** | **8.7** | **4.0** |
| Abborrtjärn | 64.1148 | 18.6986 | 366 | 11.0 | | 0.8 | 0.16 | 264 | 13.5 | 16078 |  | 7.0 | 1.9 | 20.0 | 12.8 | 7.2 | 5.9 | 10.0 | 4.6 |
| AT3 | 64.4785 | 19.4430 | 239 | 3.8 | | 0.5 | 0.12 | 254 | 8.1 | 3304 |  | 6.9 | 0.4 | 17.1 | 13.7 | 3.4 | 9.4 | 12.0 | 6.4 |
| AT4 | 64.4818 | 19.4262 | 238 | 4.3 | | 0.5 | 0.13 | 251 | 5.2 | 4748 |  | 6.8 | 0.6 | 16.8 | 13.8 | 3 | 2.4 | 13.0 | 7.5 |
| Holmtjärn | 63.9972 | 18.7091 | 299 | 9.7 | | 0.8 | 0.16 | 341 | 13.5 | 13163 |  | 7.0 | 2.0 | 19.6 | 13.0 | 6.6 | 3.8 | 7.5 | 3.1 |
| Lilla Björntjärn | 63.9158 | 18.8610 | 334 | 16.1 | | 0.4 | 0.41 | 532 | 33.9 | 9129 |  | 5.3 | 3.2 | 19.6 | 12.8 | 6.8 | 1.5 | 8.3 | 4.6 |
| Rengårdstjärnen | 64.1197 | 18.7803 | 223 | 8.0 | | 0.5 | 0.13 | 291 | 4.0 | 14777 |  | 6.8 | 1.1 | 22.5 | 12.3 | 10.2 | 4.9 | 5.3 | 2.6 |
| Snottertjärn | 64.1220 | 18.7848 | 221 | 10.2 | | 0.6 | 0.09 | 346 | 10.8 | 7995 |  | 7.2 | 1.2 | 17.3 | 13.5 | 3.8 | 2.5 | 8.3 | 2.0 |
| Jengrästjärnen | 64.4628 | 19.2905 | 264 | 10.1 | | 1.9 | 0.18 | 291 | 5.5 | 2288 |  | 7.3 | 1.2 | 22.8 | 16.4 | 6.4 | 5.8 | 13.6 | 3.3 |
| Gådatjärnen | 64.5086 | 19.4353 | 233 | 14.6 | | 1.4 | 0.42 | 351 | 5.5 | 2334 |  | 6.7 | 2.0 | 23 | 16.6 | 6.4 | 7.2 | 8.5 | 2.0 |
| Lljusv. 1S | 64.0918 | 18.9312 | 266 | 7.4 | | 1.4 | 0.94 | 325 | 18.2 | 1027 |  | 6.1 | 1.7 | 20 | 16.5 | 3.5 | 0.6 | 9.1 | 4.0 |
| Lljusv. 2N | 64.0934 | 18.9288 | 268 | 8.2 | | 1.5 | 0.97 | 262 | 15.0 | 1027 |  | 6.1 | 2.1 | 19.8 | 16.5 | 3.3 | 1.1 | 9.5 | 5.5 |
| Övre Björntjärn | 64.1231 | 18.7805 | 334 | 17.7 | | 2.0 | 1.71 | 451 | 16.7 | 12876 |  | 5.6 | 4.2 | 19.5 | 15.3 | 4.2 | 4.8 | 8.0 | 4.0 |
| Nedre Björntjärn | 64.1221 | 18.7848 | 334 | 16.7 | | 1.9 | 1.38 | 423 | 14.7 | 12876 |  | 6.0 | 4.1 | 20.1 | 15.3 | 4.8 | 3.2 | 9.7 | 6.0 |
| Lapptjärn | 64.2375 | 18.7908 | 257 | 11.4 | | 2.0 | 1.35 | 295 | 8.2 | 9953 |  | 6.0 | 2.2 | 20.9 | 15.8 | 5.1 | 2.0 | 6.5 | 2.5 |
| Mångstentjärn | 64.2505 | 18.7616 | 258 | 10.2 | | 2.8 | 1.84 | 342 | 9.7 | 9790 |  | 6.1 | 2.1 | 20.3 | 15.8 | 4.5 | 1.8 | 9.7 | 5.3 |
| Nästjärn | 64.1594 | 18.7783 | 277 | 6.8 | | 2.1 | 1.11 | 289 | 6.7 | 10646 |  | 6.3 | 1.3 | 20.5 | 15.8 | 4.7 | 1.0 | 10.4 | 4.2 |
| Fisklösan | 64.1501 | 18.8006 | 264 | 6.7 | | 1.8 | 1.06 | 257 | 8.2 | 10693 |  | 6.2 | 1.1 | 20.8 | 15.8 | 5 | 1.7 | 7.8 | 2.1 |
| Lillsjöliden | 63.8448 | 18.6168 | 318 | 13.9 | | 2.1 | 1.36 | 375 | 15.8 | 5626 |  | 6.2 | 3.0 | 15.8 | 13.1 | 2.7 | 0.8 | 5.2 | 3.8 |
| Struptjärn | 64.0233 | 19.4895 | 236 | 16.3 | | 2.3 | 1.99 | 404 | 31.4 | 3438 |  | 5.4 | 3.0 | **17.2** | 13.7 | **3.5** | 3.1 | 5.8 | 3.8 |
| Stortjärn | 64.2616 | 19.7635 | 286 | 19.5 | | 1.9 | 2.24 | 415 | 15.5 | 3128 |  | 4.8 | 4.1 | 17.2 | 13.4 | 3.8 | 3.9 | 6.7 | 2.7 |
| **Subarctic** |  |  | **582** | **5.8** | | **1.7** | **1.33** | **180** | **4.6** | **1262** |  | **5.9** | **1.1** | **17.8** | **10.5** | **7.4** | **7.0** | **8.5** | **2.6** |
| Hägglidstjärn | 63.6231 | 12.2250 | 655 | 4.4 | | 1.4 | 1.15 | 143 | 4.7 | 1283 |  | 5.8 | 0.9 | 17.1 | 10.2 | 6.9 | 4.0 | 8.5 | 2.7 |
| Vargtjärn | 63.6216 | 12.2690 | 578 | 6.6 | | 1.4 | 1.04 | 195 | 5.0 | 1092 |  | 6.0 | 1.1 | 17.7 | 10.6 | 7.1 | 11.3 | 14.5 | 3.9 |
| Lång-Björsjön | 63.6057 | 12.2655 | 580 | 5.0 | | 1.6 | 1.29 | 165 | 3.9 | 1558 |  | 5.7 | 0.9 | 18.4 | 10.5 | 7.9 | 13.2 | 10.7 | 3.0 |
| Dörrstjärnen | 63.5952 | 12.2958 | 591 | 4.6 | | 2.2 | 1.85 | 183 | 3.7 | 1495 |  | 5.7 | 0.9 | 16.5 | 10.4 | 6.1 | 4.5 | 8.7 | 2.8 |
| Gravatjärnen | 63.5846 | 12.2614 | 580 | 6.8 | | 2.6 | 1.94 | 199 | 4.9 | 1086 |  | 6.0 | 1.3 | 18.4 | 10.5 | 7.9 | 4.5 | 3.7 | 1.3 |
| Hästbäcktjärnen | 63.5842 | 12.2759 | 580 | 7.2 | | 1.0 | 0.72 | 194 | 5.2 | 1055 |  | 6.0 | 1.4 | 18.7 | 10.5 | 8.2 | 4.7 | 4.8 | 1.8 |
| **Arctic** |  |  | **633** | **5.4** | | **1.4** | **0.28** | **223** | **10.2** | **8980** |  | **6.9** | **0.6** | **14.1** | **9.9** | **4.1** | **8.3** | **8.3** | **2.9** |
| Almberga | 68.3333 | 19.1578 | 382 | 4.1 | | 1.9 | 0.18 | 167 | 10.8 | 6137 |  | 7.4 | 0.5 | 17.9 | 11.8 | 6.1 | 5.5 | 6 | 3.2 |
| Tjabrak | 68.1717 | 19.8658 | 508 | 3.2 | | 2.2 | 0.14 | 170 | 11.8 | 6344 |  | 7.6 | 0.4 | 16 | 12.1 | 3.9 | 6.2 | 14 | 4.6 |
| Ruozutjaure | 68.2042 | 19.5661 | 710 | 2.4 | | 0.8 | 0.12 | 137 | 13.6 | 9037 |  | 7.2 | 0.5 | 12 | 12.1 | -0.1 | 3.4 | 8.5 | 2.9 |
| Vuorejaure | 68.1917 | 19.6075 | 712 | 2.8 | | 0.7 | 0.12 | 141 | 13.0 | 6773 |  | 7.1 | 0.5 | 14.4 | 11.5 | 2.9 | 4.3 | 8.5 | 2.8 |
| Erkkijärvi | 67.6567 | 21.5120 | 270 | 7.1 | | 2.2 | 0.27 | 431 | 15.9 | 8911 |  | 7.2 | 0.6 | 23.7 | 11.6 | 12.1 | 11 | 4.5 | 2.3 |
| Knivsjön | 68.2939 | 19.1112 | 865 | 2.4 | | 1.9 | 0.43 | 089 | 14.7 | 9725 |  | 5.7 | 0.4 | 8.9 | 7.7 | 1.2 | 10.9 | 10.7 | 4.5 |
| Sourra | 68.2782 | 19.0954 | 993 | 1.5 | | 1.2 | 0.35 | 080 |  | 11077 |  | 6.5 | 0.3 | 8 | 6.7 | 1.3 | 17.6 | 15.8 | 4.7 |
| Estvåjärvi | 67.5991 | 20.8978 | 376 | 13.1 | | 0.8 | 0.24 | 406 | 16.3 | 14617 |  | 6.8 | 1.5 | 16.2 | 11.2 | 5 | 2.5 | 4.3 | 1.5 |
| Solbacka | 68.3474 | 18.9118 | 410 | 9.4 | | 3.5 | 0.14 | 365 | 17.1 | 7615 |  | 7.8 | 0.3 | 14.3 | 8.7 | 5.6 | 3.8 | 5.6 | 1.8 |
| 1 | 67.6567 | 21.5120 | 270 | 8.4 | | 2.8 | 0.48 | 551 | 14.0 | 7218 |  | 6.9 | 1.0 | 18.5 | 11.2 | 7.3 | 11 | 4.3 | 2.3 |
| 2 | 67.5991 | 20.8978 | 376 | 8.8 | | 1.0 | 0.48 | 294 | 13.0 | 12757 |  | 6.4 | 1.3 | 16.3 | 10.6 | 5.7 | 2.5 | 4.5 | 1.5 |
| 3 | 67.5716 | 21.1482 | 415 | 8.6 | | 0.8 | 0.34 | 416 | 10.0 | 12411 |  | 6.5 | 1.4 | 16 | 10.4 | 5.6 | 10 | 3.5 | 0.6 |
| 4 | 68.4453 | 18.6061 | 375 | 7.3 | | **10.6** | 0.33 | 211 | 2.6 | 4297 |  | 6.7 | 0.4 | 16.2 | 12.7 | 3.5 | 10.9 | 8.5 | 3 |
| 5 | 67.9650 | 20.4610 | 445 | 6.2 | | 0.8 | 0.35 | 083 | 4.9 | 5110 |  | 7.9 | 0.7 | 15.1 | 10.6 | 4.5 | 12.6 | 14 | 3.5 |
| 6 | 68.1767 | 19.8128 | 510 | 5.4 | | 2.5 | 0.62 | 182 | 3.8 | 11172 |  | 6.6 | 0.3 | 17.4 | 10.2 | 7.2 | 5.2 | 4.4 | 1.7 |
| 10 | 68.2146 | 19.6542 | 850 | 4 | | 0.2 | 0.05 | 164 | 7.9 | 4994 |  | 6.9 | 0.8 | 14.7 | 8.3 | 6.4 | 1.4 | 6.4 | 1.8 |
| 13 | 68.2737 | 18.6925 | 1045 | 3.8 | | 1.3 | 0.27 | 152 | 6.0 | 6839 |  | 6.9 | 0.3 |  | 7.1 |  | 17.4 | 15.8 | 4.7 |
| 14 | 68.2121 | 18.7281 | 1115 | 1.0 | | 0.2 | 0.08 | 065 | 3.4 | 13607 |  | 6.7 | 0.2 | 8.7 | 6.8 | 1.9 | 17.4 | 12 | 5.3 |
| 15 | 68.2374 | 18.8258 | 1140 | 3.0 | | 1.2 | 0.26 | 127 | 5.3 | 11982 |  | 6.1 | 0.3 | **0.3** | 6.4 | **-6.1** | 3.8 | 6.8 | 1.9 |

Table S3: ANOVAs testing differences in water physico-chemistry among the biomes.
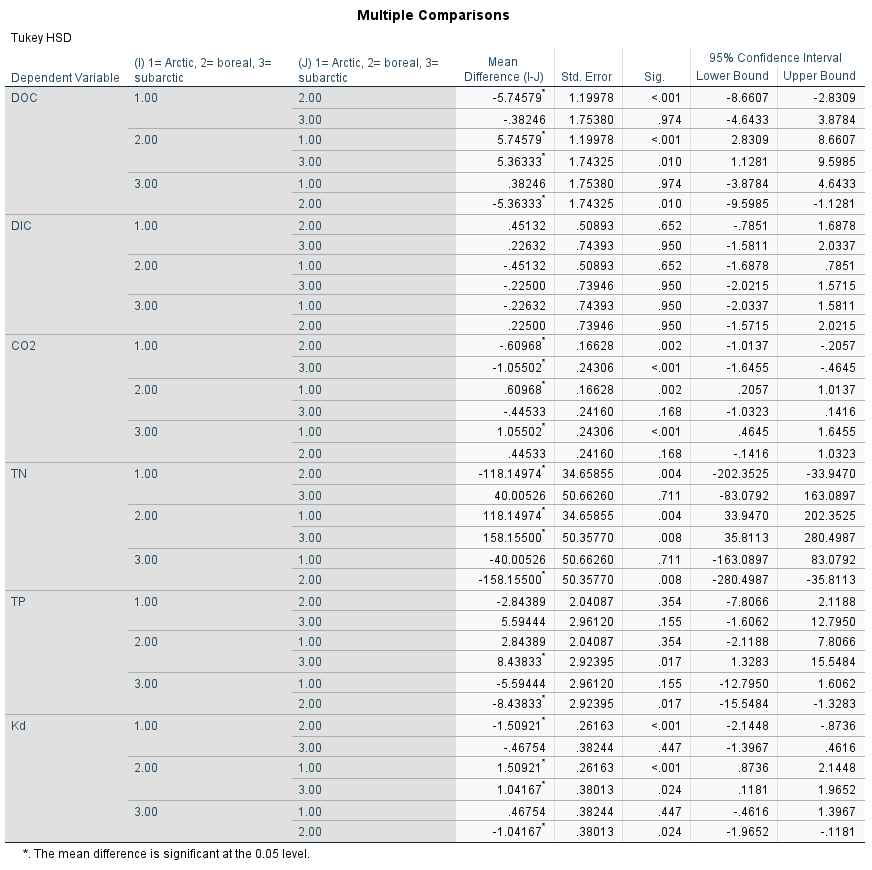


Table S4: PLS statistics of (a) GPP_z,max_ rates, and (b) GPP_lake-average_ with VIP statistics (normal font) and their loadings (italic font) per component and included variable. Tables also show the explained variance of each component, and the sum (in bold and italic font). Abbreviations are as follows: DOC= dissolved organic carbon (mg·L^-1^), DIC= dissolved inorganic carbon (mg·L^-1^), TN= total nitrogen (µg·L^-1^), TP= total phosphorus (µg·L^-1^), T_depth_= temperature at depth (°C), PAR_depth_= daily incoming PAR at depth (W·m^-2^), Area= lake surface area (hectare), z_avg_= average lake depth (m), z_max_= maximum lake depth (m), z_euph_= euphotic depth (m), T_air_= previous monthly average air temperature (°C), T_water_= temperature at 0.2m depth (°C), PAR= daily incoming PAR at surface (W·m^-2^).

1. GPP_z,max_ rates

| Component | CO2 | DOC | DIC | TN | TP | Tdepth | PARdepth |
| --- | --- | --- | --- | --- | --- | --- | --- |
| 1 | 1.70 | 1.29 | 0.87 | 0.79 | 0.48 | 0.91 | 0.04 |
| 2 | 1.67 | 1.20 | 0.81 | 0.92 | 0.52 | 0.87 | 0.50 |
| 1 | 0.46 | 0.52 | 0.37 | 0.47 | 0.26 | 0.41 | 0.13 |
| 2 | 0.52 | -0.24 | 0.17 | -0.51 | -0.47 | -0.12 | -0.56 |
|  |  | **Boreal** | |  |  |  |  |
| 1 | 1.823 | 0.788 | 1.634 | 0.457 | 0.367 | 0.134 | 0.15 |
| 2 | 1.671 | 0.877 | 1.493 | 0.696 | 0.389 | 0.122 | 0.749 |
| 1 | 0.619 | 0.467 | 0.57 | 0.359 | 0.205 |  | 0.189 |
| 2 | 0.118 | -0.43 | 0.292 | -0.59 | -0.47 |  | -0.54 |
|  |  | **Subarctic (Jämtland)** | | | |  |  |
| 1 | 0.981 | 1.256 | 0.782 | 0.735 | 1.07 | 1.339 | 0.61 |
| 2 | 0.899 | 1.134 | 0.748 | 0.664 | 1.016 | 1.279 | 1.112 |
| 1 | -0.32 | 0.489 | -0.25 | 0.388 | 0.436 | 0.481 | 0.19 |
| 2 | -0.56 | -0.32 | -0.55 | -0.51 | 0.178 | -0.1 | -0.59 |
|  |  | **Arctic (Abisko)** | | |  |  |  |
| 1 | 0.384 | 1.285 | 1.24 | 1.179 | 1.164 | 0.807 | 0.519 |
| 2 | 0.593 | 1.201 | 1.263 | 1.155 | 1.11 | 0.898 | 0.49 |
| 1 | 0.234 | 0.467 | 0.372 | 0.513 | 0.395 | 0.397 | 0.212 |
| 2 | -0.63 | -0.2 | 0.523 | -0.19 | 0.508 | -0.41 | -0.36 |

| **Component** | **CO_2_** | **DOC** | **DIC** | **TN** | **TP** | **T_depth_** | **PAR_depth_** | **Expl. var** |
| --- | --- | --- | --- | --- | --- | --- | --- | --- |
| **All lakes** | | | | | | | | |
| 1 | 1.697 | 1.293 | 0.874 | 0.792 | 0.476 | 0.909 | 0.038 | 39.6 |
| 2 | 1.673 | 1.196 | 0.813 | 0.919 | 0.515 | 0.868 | 0.496 | 22.0 |
| *1* | *0.464* | *0.523* | *0.365* | *0.465* | *0.26* | *0.411* | *0.132* | ***61.6*** |
| *2* | *0.518* | *-0.24* | *0.167* | *-0.51* | *-0.47* | *-0.12* | *-0.56* |  |
| **Boreal** | | | | | | | | |
| 1 | 1.823 | 0.788 | 1.634 | 0.457 | 0.367 | 0.134 | 0.15 | 38.0 |
| 2 | 1.671 | 0.877 | 1.493 | 0.696 | 0.389 | 0.122 | 0.749 | 24.6 |
| *1* | *0.619* | *0.467* | *0.57* | *0.359* | *0.205* |  | *0.189* | ***62.6*** |
| *2* | *0.118* | *-0.43* | *0.292* | *-0.59* | *-0.47* |  | *-0.54* |  |
| **Subarctic** | | | | | | | | |
| 1 | 0.981 | 1.256 | 0.782 | 0.735 | 1.070 | 1.339 | 0.610 | 54.3 |
| 2 | 0.899 | 1.134 | 0.748 | 0.664 | 1.016 | 1.279 | 1.112 | 12.1 |
| *1* | *-0.322* | *0.489* | *-0.251* | *0.388* | *0.436* | *0.481* | *0.190* | ***66.4*** |
| *2* | *-0.562* | *-0.315* | *-0.546* | *-0.508* | *0.178* | *-0.103* | *-0.587* |  |
| **Arctic** | | | | | | | | |
| 1 | 0.384 | 1.285 | 1.24 | 1.179 | 1.164 | 0.807 | 0.519 | 46.0 |
| 2 | 0.593 | 1.201 | 1.263 | 1.155 | 1.11 | 0.898 | 0.49 | 12.4 |
| *1* | *0.234* | *0.467* | *0.372* | *0.513* | *0.395* | *0.397* | *0.212* | ***58.4*** |
| *2* | *-0.630* | *-0.200* | *0.523* | *-0.190* | *0.508* | *-0.410* | *-0.360* |  |

1. GPP_lake-average_. VIP scores (normal font) of the first two components and their loadings (italic font). Expl. Var= explained variance per component, and total (bold).

| **Comp** | **CO_2_** | **DOC** | **DIC** | **TN** | **TP** | **PAR** | **Kd** | **Area** | **Z_max_** | **Z_avg_** | **Z_euph_** | **Alt.** | **T_air_** | **T_water_** | **DOC:TN** | **DOC:TP** | **DOC:DIC** | **Expl.var** |
| --- | --- | --- | --- | --- | --- | --- | --- | --- | --- | --- | --- | --- | --- | --- | --- | --- | --- | --- |
| **All lakes** | | | | | | | | | | | | | | | | | |  |
| 1 | 1.75 | 0.52 | 1.06 | 0.41 | 0.02 | 0.77 | 0.95 | 1.25 | 0.36 | 0.67 | 1.31 | 1.07 | 1.92 | 1.22 | 0.46 | 0.67 | 0.24 | 33.6 |
| 2 | 1.53 | 1.10 | 0.94 | 0.88 | 0.52 | 0.75 | 0.98 | 1.06 | 0.72 | 0.75 | 1.13 | 1.02 | 1.62 | 1.05 | 0.66 | 0.72 | 0.90 | 15.7 |
| *1* | *0.31* | *0.34* | *0.18* | *0.27* | *0.12* | *-0.10* | *0.35* | *-0.25* | *0.33* | *0.27* | *-0.39* | *-0.37* | *0.39* | *0.36* | *0.23* | *0.26* | *0.13* | ***49.3*** |
| *2* | *0.18* | *-0.37* | *0.22* | *-0.38* | *-0.27* | *-0.33* | *-0.24* |  |  |  | *0.20* | *0.14* |  |  | *-0.16* | *-0.12* | *-0.47* |  |
| **Boreal** | | | | | | | | | | | | | | | | | |  |
| 1 | 1.40 | 0.61 | 1.72 | 0.62 | 0.16 | 0.66 | 0.11 | 1.57 | 0.29 | 0.12 | 0.61 | 0.26 | 1.86 | 0.66 | 0.21 | 0.45 | 1.93 | 16.4 |
| 2 | 1.37 | 0.62 | 1.70 | 0.60 | 0.43 | 0.66 | 0.13 | 1.57 | 0.30 | 0.14 | 0.62 | 0.29 | 1.83 | 0.68 | 0.28 | 0.60 | 1.89 | 13.1 |
| *1* | *0.34* | *-0.11* | *0.46* | *-0.15* | *-0.11* | *-0.19* |  | *-0.32* |  | *0.23* | *-0.12* | *0.31* | *0.49* | *0.12* | *-0.23* | *-0.64* | *-0.48* | ***29.5*** |
| *2* |  | *-0.13* | *-0.24* | *0.17* | *0.56* | *0.16* |  | *-0.49* |  |  | *-0.10* |  | *-0.19* | *-0.25* |  |  |  |  |
| **Subarctic** | | | | | | | | | | | | | | | | | |  |
| 1 | 1.63 | 0.87 | 1.57 | 0.25 | 0.55 | 0.26 | 0.66 | 1.14 | 0.38 | 0.32 | 0.63 | 0.85 | 0.92 | 1.36 | 1.15 | 0.84 | 1.69 | 42.0 |
| 2 | 1.50 | 0.95 | 1.51 | 0.68 | 0.81 | 0.89 | 0.81 | 1.15 | 0.45 | 0.45 | 0.84 | 0.75 | 0.83 | 1.19 | 1.06 | 0.75 | 1.49 | 28.0 |
| *1* | *-0.29* | *0.34* | *-0.24* | *0.21* | *0.29* | *-0.26* | *0.29* | *0.14* | *0.37* | *0.38* | *-0.30* | *-0.22* | *0.27* | *0.34* | *0.35* | *0.24* | *0.38* | ***70.0*** |
| *2* | *-0.24* | *-0.27* | *-0.31* | *-0.30* | *-0.21* | *0.30* | *-0.34* | *0.33* |  |  | *0.34* |  | *-0.12* | *-0.14* | *-0.14* | *-0.18* |  |  |
| **Arctic** | | | | | | | | | | | | | | | | | |  |
| 1 | 0.78 | 1.23 | 2.13 | 1.43 | 1.54 | 0.03 | 0.21 | 0.27 | 1.04 | 0.59 | 0.13 | 1.33 | 0.54 | 1.06 | 0.24 | 0.28 | 1.02 | 31.3 |
| 2 | 0.68 | 1.09 | 1.96 | 1.27 | 1.35 | 0.04 | 0.97 | 0.40 | 0.96 | 0.73 | 0.98 | 1.23 | 0.74 | 0.99 | 0.21 | 0.34 | 1.18 | 22.8 |
| *1* | *0.20* | *0.36* | *0.35* | *0.41* | *0.34* | *-0.11* | *0.20* | *-0.15* | *-0.33* | *-0.28* | *-0.22* | *-0.43* | *0.28* | *0.35* | *-0.48* | *-0.16* | *0.13* | ***54.1*** |
| *2* |  | *-0.24* | *0.34* | *-0.13* |  |  | *-0.44* | *0.16* | *0.18* | *0.28* | *0.42* | *0.15* | *-0.17* | *-0.16* |  |  | *-0.20* |  |

## Table S5: Pearson correlations between physio chemical and bathymetry variables and a) pelagic GPP_z,max_ rates and b) pelagic GPP_lake average_. Abbreviations are as follows: PAR_depth_= PAR at depth, Temp.= temperature (°C), DOC= dissolved organic carbon (mg·L^-1^), DIC= dissolved inorganic carbon (mg·L^-1^), TN= total nitrogen (µg·L^-1^), TP= total phosphorus (µg·L^-1^), T_air_= air temperature (°C), T_depth_= temperature at depth (°C), z_max_= maximum lake depth, z_avg_= average lake depth (m), z_euph_= euphotic depth, Alt.=Altitude (m above sea level).

1. Pelagic GPP_z,max_ rates (in mg C·m^-3^·day^-1^) and physio chemical and bathymetry variables. Abbreviations are as follows: CO_2_= carbon dioxide in lake water (mg·L^-1^), DOC= dissolved organic carbon (mg·L^-1^), DIC= dissolved inorganic carbon (mg·L^-1^), TN= total nitrogen (mg·L^-1^), TP= total phosphorus (µg·L^-1^), T_depth_ = temperature at depth (°C), PAR_depth_= daily incoming PAR at depth (W·m^-2^) ^a^ denotes log transformation of the variable, and all correlations displayed have a p<0.05

|  | GPP_z,max_ ^a^ | CO_2_ | DOC | DIC | TN | TP | T_depth_ | PAR_depth_ |
| --- | --- | --- | --- | --- | --- | --- | --- | --- |
| GPP_z,max_^a^ | 1 | .75 | .65 |  | .48 |  | .62 |  |
| CO_2_ | .75 | 1 | .47 |  |  |  |  |  |
| DOC | .65 | .47 | 1 |  | .81 | .56 | .52 | .30 |
| DIC |  |  |  | 1 |  |  |  |  |
| TN | .48 |  | .81 |  | 1 | .62 | .57 | .41 |
| TP |  |  | .56 |  | .62 | 1 |  |  |
| T_depth_ | .62 |  | .52 |  | .57 |  | 1 |  |
| PAR_depth_ |  |  | .30 |  | .41 |  |  | 1 |

1. Pelagic GPP_lake average_ (in mg C·m^-2^·day^-1^) and physio chemical and bathymetry variables. Abbreviations are as follows: GPP _lake-avg_.= GPP_lake-average_, CO_2_= carbon dioxide in lake water (mg·L^-1^), DOC= dissolved organic carbon (mg·L^-1^), DIC= dissolved inorganic carbon (mg·L^-1^), TN= total nitrogen (mg·L^-1^), TP= total phosphorus (µg·L^-1^), PAR= daily incoming PAR at surface (W·m^-2^), Area= lake surface area (m^2^), z_max_= maximum lake depth (m), z_avg_= average lake depth (m), Alt.= lake altitude (m), T_air_ = previous monthly average air temperature (°C), T_water_ = temperature at 0.2m (°C). ^a^ denotes log transformation of the variable, and all correlations displayed have a p<0.05.

|  | GPP _lake-avg_^a^ | CO_2_ | DOC | DIC | TN | TP | PAR | Kd | Area | z_max_ | z_avg_ | z_euph_ | Alt. | T_air_ | T_water_ | DOC: TN^a^ | DOC: TP^a^ | DOC: DIC^a^ |
| --- | --- | --- | --- | --- | --- | --- | --- | --- | --- | --- | --- | --- | --- | --- | --- | --- | --- | --- |
| GPP _lake-avg_^a^ | 1 | .73 | .46 |  | .32 |  | -.31 | .50 | -.30 |  |  | -.51 | -.42 | .56 | .44 | .39 | .34 |  |
| CO_2_ | .73 | 1 | .47 |  |  |  | -.30 | .62 |  |  |  | -.52 |  | .33 |  | .43 | .34 |  |
| DOC | .46 | .47 | 1 |  | .81 | .56 |  | .91 | -.41 | -.34 |  | -.71 | -.62 | .56 | .49 | .59 | .41 | .56 |
| DIC |  |  |  | 1 |  |  |  |  |  |  |  |  |  |  |  |  | .33 | -.56 |
| TN | .32 |  | .81 |  | 1 | .62 |  | .67 | -.37 | -.47 |  | -.62 | -.71 | .51 | .54 |  |  | .48 |
| TP |  |  | .56 |  | .62 | 1 |  | .55 | -.35 |  |  | -.33 | -.32 |  |  |  | -.43 |  |
| PAR | -.31 | -.30 |  |  |  |  | 1 |  |  |  |  |  |  |  |  |  |  |  |
| Kd | .50 | .62 | .91 |  | .67 | .55 |  | 1 | -.44 |  |  | -.75 | -.49 | .57 | .41 | .54 |  | .53 |
| Area | -.30 |  | -.41 |  | -.37 | -.35 |  | -.44 | 1 | .47 |  | .55 | .50 | -.56 | -.31 |  |  | -.39 |
| z_max_ |  |  | -.34 |  | -.47 |  |  |  | .47 | 1 | .69 | .32 |  |  |  |  |  |  |
| z_avg_ |  |  |  |  |  |  |  |  |  | .69 | 1 |  |  |  |  |  |  |  |
| z_euph_ | -.51 | -.52 | -.71 |  | -.62 | -.33 |  | -.75 | .55 | .32 |  | 1 | .69 | -.69 | -.71 | -.52 | -.42 | -.59 |
| Alt. | -.42 |  | -.62 |  | -.71 | -.32 |  | -.49 | .50 |  |  | .69 | 1 | -.83 | -.83 |  | -.38 | -.38 |
| T_air_ | .56 | .33 | .56 |  | .51 |  |  | .57 | -.56 |  |  | -.69 | -.83 | 1 | .75 |  | .31 | .32 |
| T_water_ | .44 |  | .49 |  | .54 |  |  | .41 | -.31 |  |  | -.71 | -.83 | .75 | 1 |  | .47 | .36 |
| DOC:  TN^a^ | .39 | .43 | .59 |  |  |  |  | .54 |  |  |  | -.52 |  |  |  | 1 | .55 | .30 |
| DOC:  TP^a^ | .34 | .34 | .41 | .33 |  | -.43 |  |  |  |  |  | -.42 | -.38 | .31 | .47 | .55 | 1 |  |
| DOC:  DIC^a^ |  |  | .56 | -.56 | .48 |  |  | .53 | -.39 |  |  | -.59 | -.38 | .32 | .36 | .30 |  | 1 |

Figures


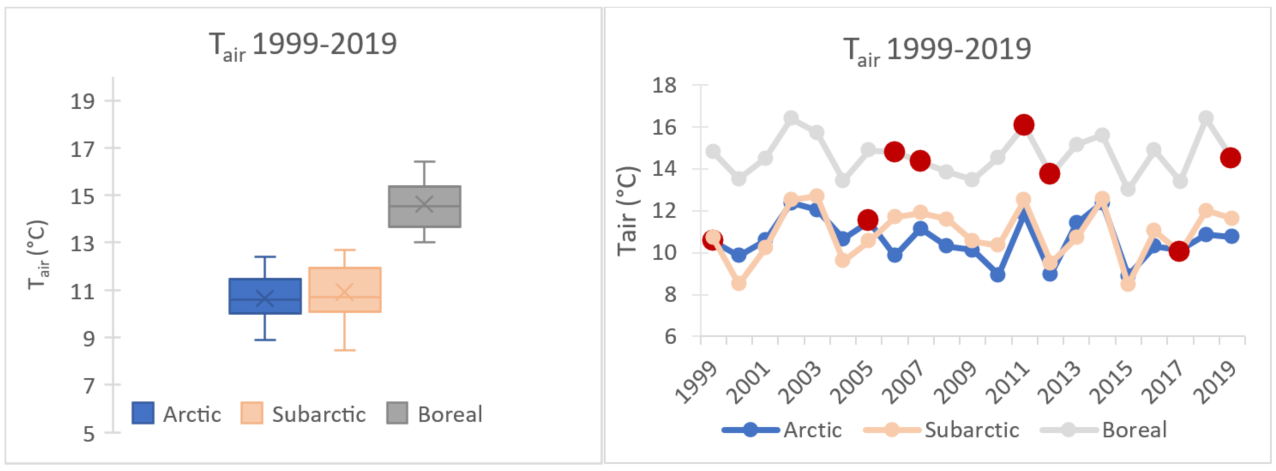


(a)

(b)

Figure S1: Average monthly temperatures in June and July for the years 1999-2019 for the Arctic, subarctic and boreal biomes summarized in (a) a boxplot and (b) as variation over time, with sampling occasions indicated in red.

Figure S2: Correlation between Kd (m^-1^) and DOC (mg·L^-1^).


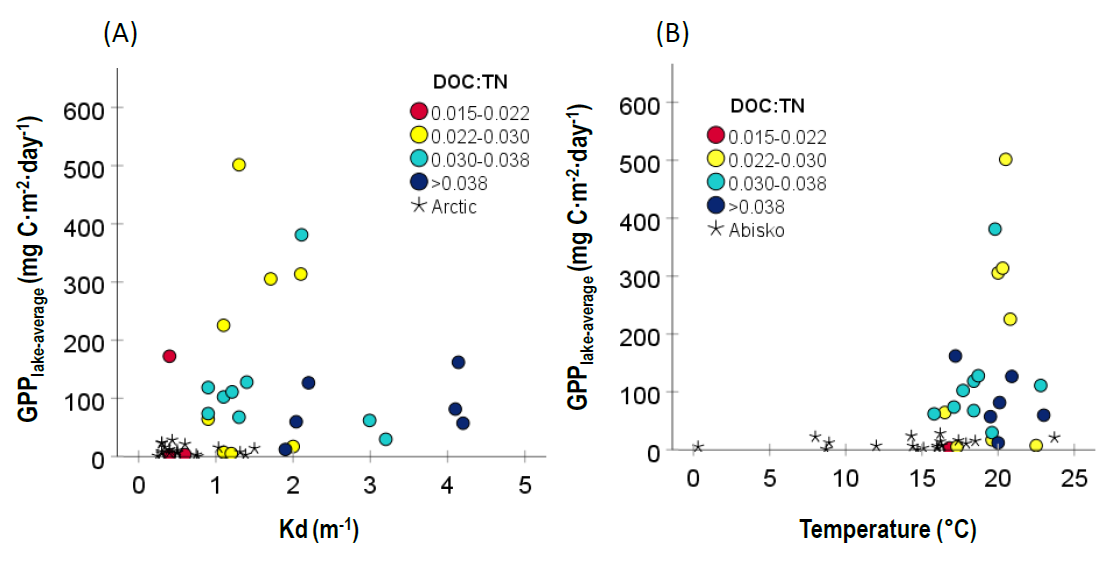


Figure S3: GPP_lake-average_ plotted in different categories DOC:TN ratios against (a) Kd (m^-1^), and (b) water temperature at 0.2m depth (°C). Lakes from the Arctic biome are plotted as a separate category (star shaped).
